# Genomic hotspots of chromosome rearrangements explain conserved synteny despite high rates of chromosome evolution in a holocentric lineage

**DOI:** 10.1111/mec.17086

**Published:** 2023-07-24

**Authors:** Marcial Escudero, André Marques, Kay Lucek, Andrew L. Hipp

**Affiliations:** ^1^ Department of Plant Biology and Ecology University of Seville Sevilla Spain; ^2^ Department of Chromosome Biology Max Planck Institute for Plant Breeding Research Cologne Germany; ^3^ Institute of Biology University of Neuchâtel Neuchâtel Switzerland; ^4^ The Morton Arboretum Lisle Illinois USA

**Keywords:** *Carex*, centromere, comparative genomics, Cyperaceae, RAD‐seq loci, repetitive DNA

## Abstract

Holocentric organisms, unlike typical monocentric organisms, have kinetochore activity distributed along almost the whole length of the chromosome. Because of this, chromosome rearrangements through fission and fusion are more likely to become fixed in holocentric species, which may account for the extraordinary rates of chromosome evolution that many holocentric lineages exhibit. Long blocks of genome synteny have been reported in animals with holocentric chromosomes despite high rates of chromosome rearrangements. Nothing is known from plants, however, despite the fact that holocentricity appears to have played a key role in the diversification of one of the largest angiosperm genera, *Carex* (Cyperaceae). In the current study, we compared genomes of *Carex* species and a distantly related Cyperaceae species to characterize conserved and rearranged genome regions. Our analyses span divergence times ranging between 2 and 50 million years. We also compared a *C. scoparia* chromosome‐level genome assembly with a linkage map of the same species to study rearrangements at a population level and suppression of recombination patterns. We found longer genome synteny blocks than expected under a null model of random rearrangement breakpoints, even between very distantly related species. We also found repetitive DNA to be non‐randomly associated with holocentromeres and rearranged regions of the genome. The evidence of conserved synteny in sedges despite high rates of chromosome fission and fusion suggests that conserved genomic hotspots of chromosome evolution related to repetitive DNA shape the evolution of recombination, gene order and crossability in sedges. This finding may help explain why sedges are able to maintain species cohesion even in the face of high interspecific chromosome rearrangements.

## 1 INTRODUCTION

Chromosome fissions and fusions may affect reproductive barriers and drive speciation either directly, through hybrid dysfunction or recombination suppression, or indirectly, through reinforcing non‐chromosomal speciation (de Vos et al., 2020; Fishman et al., 2014; Raskina et al., 2008; Rieseberg, 2001; Twyford et al., 2015). These rearrangements are frequently associated with rearrangement hotspots in genomic regions that entail the non‐random breakage of chromosomes. Microsatellites or AT‐rich minisatellite arrays (Sutherland et al., 1998), other repetitive DNA (including transposable elements) and rDNA have been suggested to cause such chromosome instability (Raskina et al., 2008). Rearrangement hotspots are frequent in different lineages of animals (Ruiz‐Herrera et al., 2002; Toledo et al., 2000), including humans (Hellman et al., 2002; Kaufmann & Reiss, 1999). To a lesser degree, they are present in plants as well (Grabowska‐Joachimiak et al., 2015; Huang et al., 2008, 2009; Lan et al., 2016; Li et al., 2017).

All eukaryotic organisms possess either monocentric or holocentric chromosomes. In monocentric chromosomes, kinetochore activity is concentrated in a single centromere. In contrast, holocentric chromosomes harbour multiple centromere‐like units along almost their whole length, resulting in a line‐like holocentromere in the condensed chromosomes (Hipp et al., 2009, 2013; Marques et al., 2015). Holocentric chromosomes are estimated to be present in 15%–20% of eukaryote species and have independently evolved at least 19 times in both plants and animals (Escudero, Márquez‐Corro, & Hipp, 2016; Melters et al., 2012).

Fission and fusion events can destabilize monocentric chromosomes. Chromosome fragments that either lack centromeres due to fission or have two centromeres due to fusion are likely to suffer difficulties during segregation, often resulting in unviable gametes. Fragmented holocentric chromosomes, however, due to their diffuse centromere‐like structures, appear to segregate normally during meiosis and are inherited in Mendelian fashion (Faulkner, 1972; Luceño, 1993). Holocentric chromosomes therefore allow rapid evolution of chromosome rearrangements via fission and fusion (Hipp et al., 2009). As a consequence, holocentromeres have the potential to reduce or eliminate the underdominance of chromosome rearrangements, at least for heterozygotes between similar cytotypes within species, allowing them to establish and become fixed at a higher rate than in organisms with monocentric chromosomes (Lucek et al., 2022).

Besides the holocentric nematode model organism *C. elegans* and its close relatives, which behave as monocentric species for many genomic and meiotic features (Márquez‐Corro et al., 2019), comparative genomics of species with holocentric chromosomes is still very limited, but the few studies that have been done are suggestive that holocentricity may significantly shape the evolution of biodiversity. In Lepidoptera, the largest holocentric clade, long synteny blocks are conserved in spite of high rates of chromosome evolution (ca. 2 chromosome breaks Mb^−1^ Ma^−1^, d'Alençon et al., 2010). Interestingly, comparative and functional genomics show unusual features common to organisms with holocentric chromosomes (at least for Lepidoptera and Cyperaceae), such as a uniform GC content and gene distribution along chromosomes (Hofstatter et al., 2022; Mandrioli & Carlo Manicardi, 2012). However, holocentric clades may exhibit unique features as well. For example, unlike organisms with monocentric chromosomes, in which recombination decreases towards the centromeres and increases towards the telomeres, recombination rates are relatively uniformly distributed in Lepidoptera (Haenel et al., 2018; but not that homogeneous in holocentric plants, Hofstatter et al., 2022). Interestingly, extensive end‐to‐end chromosome fusions (associated with LTR retroelements) play an important role in karyotype evolution and structural diploidization in *Rhynchospora* beak‐sedges despite high conservation of synteny (Hofstatter et al., 2022). More studies are needed to determine the correlates and consequences of holocentricity, and how universal these may be.

Moreover, genome rearrangements bear a potential fitness cost even in holocentric organisms, as rearrangements may affect gene function or expression. At its worst, chromosome evolution could be a deal with the Devil for holocentric organisms, if increased rates of recombination result in both increased diversification rates and decreased individual fitness. There is some evidence that sedges may have accepted the trade. Despite the available evidence suggesting homogeneous distribution of repetitive DNA and density of genes in holocentric species (Haenel et al., 2018; Hofstatter et al., 2022; Mandrioli & Carlo Manicardi, 2012), repetitive DNA (LTR retroelements) is significantly associated with chromosome fusions in beak‐sedges (Hofstatter et al., 2022). This is what we expect to find if chromosome rearrangements impact fitness more strongly in gene regions than outside of them. This finding bears investigation in more lineages, searching for whether there are significant differences between rearranged and conserved genomic regions, with more repetitive DNA (TEs and satDNA) in rearranged regions and higher gene density in conserved regions. Notwithstanding that repeat regions may be involved in gene regulation, overrepresentation of rearrangement hotspots in repeat regions instead of coding regions would suggest that repeat regions serve a structural role in holocentric organisms, giving chromosomes a safe place to break or fuse.

Here we performed a comparative genomic study in the holocentric sedge genus *Carex*, one of the most diverse plant genera. We aimed to (i) reconstruct patterns of genome synteny and rearrangements in *Carex*, (ii) elucidate the patterns of satellite DNA (satDNA), transposable elements (TE) and their types (class I LTR transposons Ty1‐copia and Ty3 and class II non‐LTR LINE), and density of genes between conserved and rearranged genomic regions in *Carex*, and (iii) model the evolution of chromosome rearrangements based on estimated rates of chromosome fission and fusion. We use simulated chromosome rearrangements to assess whether patterns found in (i) and (ii) are significant relative to the null expectation of no correlation between rearrangements and genome structure, and to evaluate whether synteny blocks are more or less conserved than expected.

## 2 MATERIALS AND METHODS

### 2.1 Overview of analyses

We first performed macroevolutionary analyses of shifts of rates of chromosome evolution and reconstruction of chromosomal transitions across the phylogeny of the genus *Carex* to establish a general framework of chromosome evolution. For our comparative analyses, we used a linkage map of *Carex scoparia* and four genomes of the species *C. scoparia*, *C. cristatella*, *C. little‐dalei*, and, for family‐level comparison, *Rhynchospora breviuscula*. We performed a first set of synteny analyses comparing the linkage map of *C. scoparia* with the genomes of *C. scoparia*, *C. cristatella* and *C. little‐dalei* (we excluded the genome of *R. breviuscula* here because it is a more distantly related species from another genus, and we perceived, based on our own results, that the comparison between the linkage map of *C. scoparia* and *C. little‐dalei* genome was already hampered by homology). Then we performed a second set of synteny analyses comparing the four available genomes. We also performed a de novo repeat discovery and annotation analyses for these genomes. We compared the genomic features of conserved and rearranged genome areas (based on comparisons between the *C. scoparia* linkage map and the genomes as well as among‐genome comparisons). Finally, we quantified the number of rearrangements estimated from comparative genomics approaches and compared those estimates with the expected number of rearrangements following different models.

### 2.2 Rates of chromosome evolution in genus *Carex*

The datasets from Márquez‐Corro et al. (2021) were used in this study. Márquez‐Corro et al. (2021) used a three‐gene *Carex* phylogeny from Martín‐Bravo et al. (2019, comprising 66% of the taxonomic diversity of *Carex*). The phylogeny was pruned to keep only the species with available chromosome information (*N* = 755) and transformed to be ultrametric and fully dichotomous following Márquez‐Corro et al. (2021). Chromosome number counts were also taken from Márquez‐Corro et al. (2021), originally obtained from Rice et al. (2015) and Roalson (2008).

Bayesian analyses of macroevolutionary mixtures (BAMM 2.5; Rabosky et al., 2013; Rabosky, Donnellan, et al., 2014; Rabosky, Grundler, et al., 2014; Shi & Rabosky, 2015) were used to model evolution of diploid chromosome number on the *Carex* phylogeny. BAMM uses reversible‐jump Markov Chain Monte Carlo (MCMC) to explore a vast diversity of candidate models of trait evolution, allowing shifts in trait rates and changes over time within each regime. BAMM was run twice for ten million generations and MCMC convergence was checked with the R package “coda” (Plummer et al., 2006). The “BAMMtools” R package (Rabosky, Grundler, et al., 2014) in R (R Core Team, 2021) was used to process the results and summarize the parameters of the models with the highest posterior probabilities.

The history of chromosome number evolution was modelled on the phylogeny using ChromEvol 2.0 (Glick & Mayrose, 2014; Mayrose et al., 2010). A single model that includes all available parameters for chromosome transitions was fitted (includes eight rate parameters: constant fission and fission linearly proportional to chromosome number, constant fusion and fusion linearly proportional to chromosome number, demiduplication, duplication and chromosome base number multiplication and a base number). The inferred model parameters were used for the reconstruction of chromosome numbers and rearrangements in the phylogeny (Glick & Mayrose, 2014).

### 2.3 Genomic data

Genomic data for *C. little‐dalei* (accessions CM022079‐CM022107; Clark et al., 2016), *C. scoparia* and *C. cristatella* (Bioproject PRJNA723756; Planta et al., 2022) and *R. breviuscula* (Bioproject PRJNA784789; Hofstatter et al., 2022) were downloaded from GenBank. The linkage map of *C. scoparia* was from Escudero et al. (2018). The latter comprised linkage location of 1426 RAD‐seq loci from a linkage map of F2 individuals from a cross between an individual of *C. scoparia n* = 32 and another with *n* = 33 (Escudero et al., 2018).

### 2.4 Synteny in *Carex* using linkage map against genomes: Conserved versus rearranged genome regions

The complete 130–150 bp sequences of all RAD‐seq sites used for the *C. scoparia* linkage map were mapped to the *C. scoparia*, *C. cristatella*, and *C. little‐dalei* genomes using Bowtie 2 (Langmead & Salzberg, 2012). Two mapping datasets were analysed for *C. little‐dalei* (the most distantly related species): (i) every RAD‐seq locus that mapped to the genome, using only the best match for loci that mapped to more than one location; and (ii) only RAD‐seq loci that mapped to a single location. Mappings were visualized using RCircos (Zhang et al., 2013) package in R (R Core Team, 2021).

Sections of the *C. little‐dalei*, *C. scoparia* and *C. cristatella* genomes were then classified as (i) conserved regions when at least two consecutive RAD‐seq loci from the same *C. scoparia* linkage group were contiguously found in the *Carex* genome or (ii) rearranged regions (the section between two conserved regions from different linkage groups of *C. scoparia*) using R scripts (R Core Team, 2021). Accordingly, only chromosome rearrangements that entail fission or fusion between two different chromosomes were quantified. A third category—“single marker”—was used for solitary loci surrounded by loci from other linkage groups. “Single markers” could not be determined to be conserved or rearranged. Rearrangements not entailing chromosome number change (e.g. inversions) were not quantified because they would be more difficult to quantify when comparing linkage mapping with the genomes (especially between more distantly related species. Exceptionally, inversions were quantified within *C. scoparia*).

Patterns of recombination suppression were characterized by comparing the *C. scoparia* genome (from a population in Michigan, USA) with the linkage map of the same species (from a cross between a population from Indiana and a population from Wisconsin). Catchen et al. (2011) compared a linkage map and genome of the zebrafish and established that 10 Mb RAD‐seq gap in the physical genome of the zebrafish (4498.8 Mp) was considered as experimental support for suppression recombination. We considered large genomic sections ≥1 Mb in the *C. scoparia* genome (ca. 335 Mb in total) without any RAD‐seq hit from the linkage map as experimental support for suppression of recombination (taking into account that *C. scoparia* genome is over 10 times smaller than the zebrafish genome and that the density of the *C. scoparia* linkage map is higher than the density of the zebrafish linkage map).

### 2.5 Synteny in *Carex* (and Rhynchospora) using genomes against genomes: conserved versus rearranged genome regions

The synteny calculations between *C. little‐dalei* and *R. breviuscula*, *C. little‐dalei* and *C. cristatella*, *C. little‐dalei* and *C. scoparia* and *C. cristatella* and *C. scoparia* were performed with SyMAP v. 5.0.6 (Soderlund et al., 2006, 2011) and GENSPACE v. 1.3.0 (Lovell et al., 2022). Synteny plots were obtained using the synteny calculation blocks obtained from SyMAP and GENESPACE.

### 2.6 De novo repeat discovery and annotation

RepeatExplorer2 was used for de novo repeat discovery (Novák et al., 2020) for the three *Carex* genomes. For each species, a repeat library was obtained from the RepeatExplorer2 analysis of Illumina paired‐end reads. Clusters representing >0.005% of the genome were manually checked, and the automated annotation was corrected if needed. Contigs from the annotated clusters were used to build a repeat library. To minimize potential conflicts due to the occasional presence of contaminating sequences in the clusters, only contigs with average read depths ≥5 were included, and all regions in these contigs that had read depths <5 were masked.

Transposable element protein domains (Neumann et al., 2019) found in the assembled genomes were annotated using the DANTE tool available from the RepeatExplorer2 Galaxy portal (https://repeatexplorer‐elixir.cerit‐sc.cz/galaxy/), exploiting the REXdb database (Viridiplantae_version_3.0; Neumann et al., 2019). Tandem repeat annotations were performed using the TAREAN tool available from the RepeatExplorer2 output. Consensus monomers were then used as bait to annotate the presence and overall distribution of satellite DNA repeats in the assembled genome using the annotation tool in Geneious R9 (Kearse et al., 2012).

### 2.7 Characterization of conserved versus rearranged genome regions

Locations of TEs and satellite DNA were obtained from the aforementioned annotation and gene annotation of *Carex* genomes (Can et al., 2020; Planta et al., 2022). Once repetitive DNA (TE and satDNA) and locations of genes were characterized for the three *Carex* genomes, comparing them with the linkage mapping genome, their probability of being associated with rearranged regions was tested with generalized linear models (glm) in R (R Core Team, 2021) under a binomial distribution.

The rearrangement points when comparing *C. scoparia* and *C. cristatella* genomes were also characterized to find patterns of gene density or repetitive DNA density. To test if chromosome rearrangements are associated with differences in gene density or the number of repetitive elements, a linear mixed‐effects model was fit using restricted maximum likelihood (REML) as implemented in the *lme4* package in R. Along the genomes of *C. scoparia* and *C. cristatella*, the number of genes, centromeric DNA sequences, and repeats of different types (classII TEs, Ty3, Ty1‐Copia, class II non‐LTRs LINEs, tRNAs) were counted within 100 kb windows. For each window, the distance from the midpoint of the chromosome was calculated, and for each chromosome the number of rearrangement points of one species against the other was taken into account. Models were fitted for each species separately with the number of genes, centromeric DNA and repeats as dependent variables, respectively, and the number of rearrangements points, the log10 transformed distance of each window from the telomeric region to the chromosome midpoint as well as their interaction as fixed effects. Random effects were chromosomes and whether a window was at the 5′ or 3′ end from the chromosome midpoint.

### 2.8 Quantifying chromosome rearrangements

The rearrangements between *Carex* species estimated by chromEvol cannot be directly compared with the number of rearrangements estimated when comparing *Carex* genomes and *Carex scoparia* linkage map for at least two reasons. First, chromEvol does not reconstruct chromosome rearrangements directly, but only changes in chromosome number; by contrast, rearrangements inferred from the linkage map take into account even rearrangements that result in no change in chromosome number. Second, the power to detect rearrangements from linkage mapping data becomes more and more limited the more distantly related a species is. To expand the comparison between *Carex* genome and the linkage map, simulations were performed to estimate the number of rearrangements that could be detected. Accordingly, the number of rearrangements inferred from chomEvol was then simulated on the *Carex* genomes (Can et al., 2020), 999 times. These rearrangements were set to happen subsequently in each simulation, and the probability of fission and fusion was equal (i.e. 0.5) as even slight initial preference towards fusion or fission would end simulations always into extraordinarily small or large chromosome numbers, respectively. The probability for a fission at any point of the genome was equal, making the probability for a fission on any chromosome proportional to each chromosome size. To keep more similar chromosome sizes, the probability of fusion was set as inversely proportional to chromosome size. Consequently, a very small chromosome would have a higher probability of being fused to another chromosome. Finally, conserved versus rearranged genome areas were inferred, comparing the distribution of these simulated data against the observed data to infer whether or not the observed number of rearrangements in simulations are significantly smaller than expected when there are no constraints on where in the genome the rearrangement may happen.

In the comparison between *C. little‐dalei* and *R. breviuscula* genomes, *C. little‐dalei* and *C. cristatella* genomes, and *C. cristatella* and *C. scoparia* genomes, the numbers of inferred rearrangements were directly counted. Because whole genome sequences were compared here, all kinds of rearrangements were counted (fissions, fusions and inversions).

## 3 RESULTS

### 3.1 Rates of chromosome evolution and number of rearrangements

**FIGURE 1:**
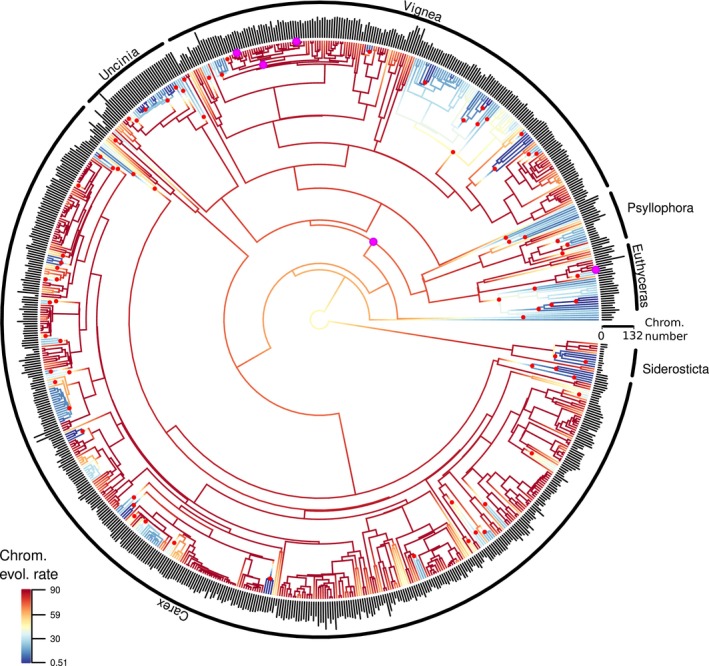
Rates of chromosome evolution in genus *Carex* inferred using BAMM. Shifts in rates of chromosome evolution with PP > 0.30 are shown in red dots (75 shifts). The locations of the species *C. scoparia*, *C. cristatella* and *C. little‐dalei* and the last common ancestor of them are shown with pink dots. Diploid chromosome numbers are shown in vertical bars in the tips of the phylogeny (from 2n = 10 the shortest bar to 2n = 132 the largest bar; see chromosome number scale). Main *Carex* clades are indicated with arcs. Rates of chromosome evolution are indicated in the branches with a gradient of colours (from 0.51 to 90 chromosomes per million years, from dark blue for the slowest rates to dark red for the fastest rates. See the legend in the bottom left corner).

BAMM estimated high heterogeneity (high number of shifts, *N* = 124, MCMC 95% HPD = 78–176) in the rate of chromosome evolution (Figure 1). The rate of chromosome evolution at the root of the phylogeny was 42.1 (MCMC 95% HPD = 38.1–61.4) chromosome number changes Ma^−1^ with a growth parameter of 0.0836 units (MCMC 95% HPD = 0.0379–0.1669), reaching a rate of 81.7 (MCMC 95% HPD = 72.9–111.3) chromosome Ma^−1^ at the tips of the phylogeny. The 124 shifts are sudden decreases of rates of chromosome evolution in more shallow clades, ranging from very low rates of evolution in some clades (<0.01 chromosomes Ma^−1^) to relatively high rates of chromosome evolution in others (8.4 chromosome Ma^−1^; Table S1; Figure 1). While reconstructing the evolution of chromosome number is not the same as reconstructing the evolutionary history of chromosome rearrangements themselves, this analysis provided insights into how rates of chromosome evolution have shifted over time and across clades. The estimated parameters of the chromEvol model suggested low rates of polyploidy and very high rates of fission and fusion, both constant rates and linearly proportional to the current haploid chromosome number. However, fission and fusion do not respond the same to chromosome number, as evidenced by the higher slope of rates of fusion regressed on chromosome number relative to rates of fission regressed on chromosome number. Both fission and fusion increase with chromosome number, but fusion responds more (Table S2). Based on the chromEvol reconstruction, 277 events of fission and fusion were inferred since *C. little‐dalei* and *C. scoparia* split (counting only along the branches separating these two species), 276 between *C. little‐dalei* and *C. cristatella* and 29 between *C. scoparia* and *C. cristatella*. Four rearrangements were estimated in the lineage of *C. scoparia*.

### 3.2 Synteny inferred from linkage markers mapped to genome assemblies

Genome synteny was assessed by pairwise comparisons of a genetic linkage map of *Carex scoparia* with each of three chromosome‐level genome assemblies that represent increasingly closely related lineages (*C. little‐dalei* as the most distantly related, *C. cristatella*, and *C. scoparia*). 547 RAD‐seq loci mapped to the *C. little‐dalei* genome (Figure S1), of which 443 (81.0%) mapped uniquely (Figure S1). The mapping results were similar for the full and uniquely‐mapped RAD‐seq datasets: each *C. little‐dalei* chromosome maps on average to only two or three *C. scoparia* linkage groups. The dataset comprising 547 loci was consequently used for further analyses. A total of 81 conserved genome regions of at least two contiguous RAD‐seq loci and 33 rearrangements were identified. In addition, 50 “single markers” occurred, i.e. isolated RAD‐seq loci surrounded by loci from a different linkage group. These single markers could not be classified as conserved or not.

1246 RAD‐seq loci mapped to the *C. cristatella* genome (Figure S1), of which 1161 (93.2%) mapped uniquely. Most *C. cristatella* chromosomes map to only one or two *C. scoparia* linkage groups (Figure S1). 73 genome regions were conserved, with 23 rearrangements and 23 single markers.

**FIGURE 2:**
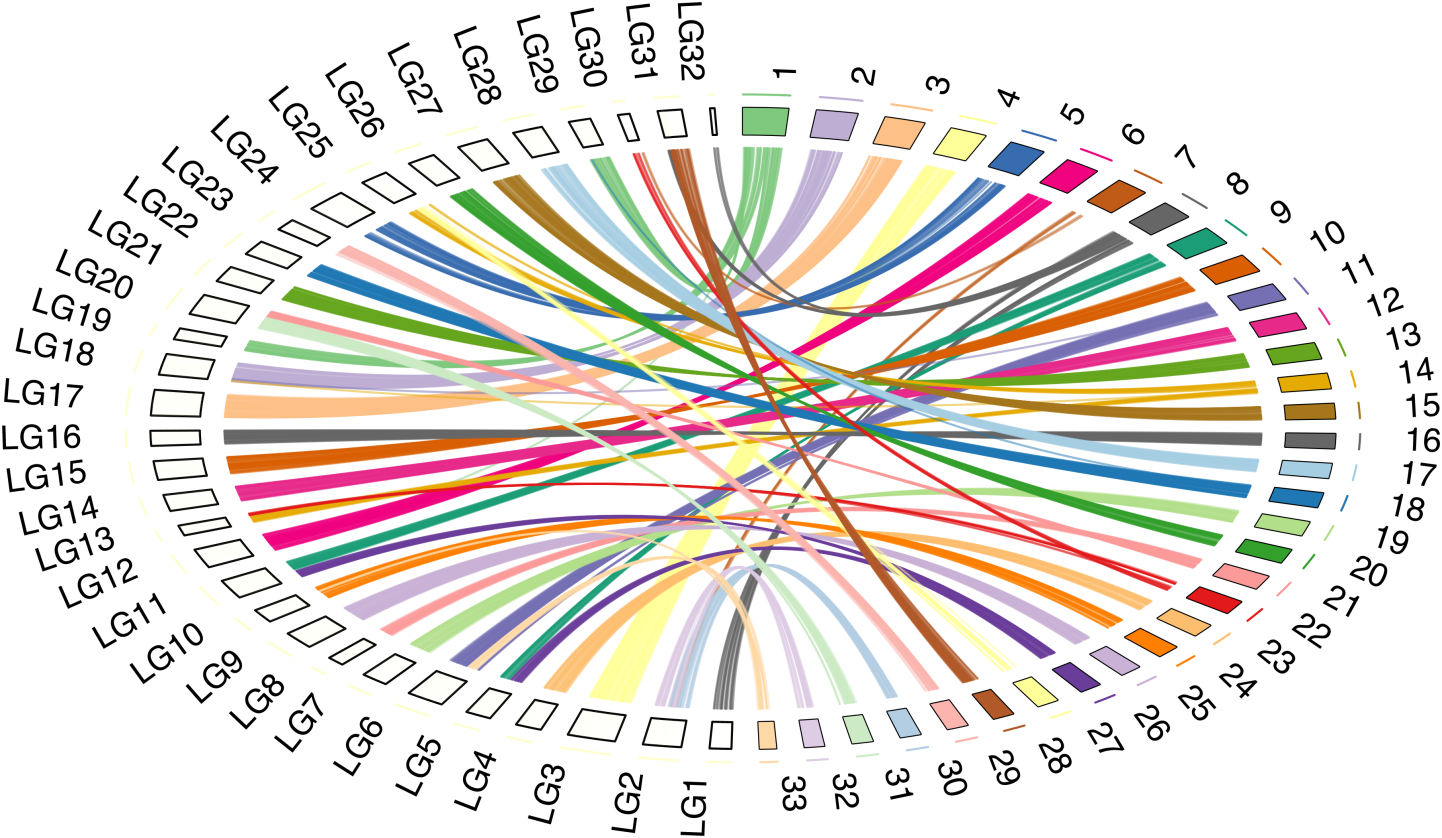
RCircos image showing *C. scoparia* chromosomes and *C. scoparia* linkage groups ideograms with data tracks for connectors link lines between both genomes. The 14 fission/fusion events, four inversion and pattern of recombination suppression as well as recombination gaps in *C. scoparia* genome can be observed.

1382 RAD‐seq loci mapped to the *C. scoparia* genome (Figure 2), of which 1206 (87.3%) mapped uniquely. There was a high degree of synteny, and on average each *C. scoparia* genome chromosome mapped to only one *C. scoparia* linkage group (Figure 2). Mapping the *C. scoparia* linkage markers to the *C. scoparia* genome assembly revealed 51 conserved genome regions, 14 rearrangements and four single markers. Interestingly, four inversions could be clearly identified for *C. scoparia*. 21 genome regions of 1 Mb or larger in *C. scoparia* lacked any RAD‐seq hit from the linkage map, representing potential regions of suppressed recombination. Most of these regions (16 out 21) bracketed chromosome rearrangements (14 fissions and fusions and four inversions) inferred in this study.

A mean of 50.54 (SD = 9.26) rearrangements were inferred by simulating the evolution of *C. scoparia* linkage groups versus *C. little‐dalei* genome (based on the number of rearrangements estimated from chromEvol). The observed number of rearrangements (*n* = 33) was significantly smaller than expected based on simulations (*p* = .031). This contrasts with a mean of 11.89 (SD = 2.65) rearrangements when simulating the evolution of *C. scoparia* linkage groups versus *C. cristatella* genome. The observed data (*n* = 23 rearrangements) was significantly higher (*p* < .001). Finally, a mean of 3.76 (SD = 1.43) rearrangements was inferred when simulating the evolution of *C. scoparia* linkage groups versus *C. scoparia*. The observed data, 14 rearrangements, was also significantly higher (*p* < .001).

### 3.3 Synteny inferred from comparative genome assemblies

**FIGURE 3:**
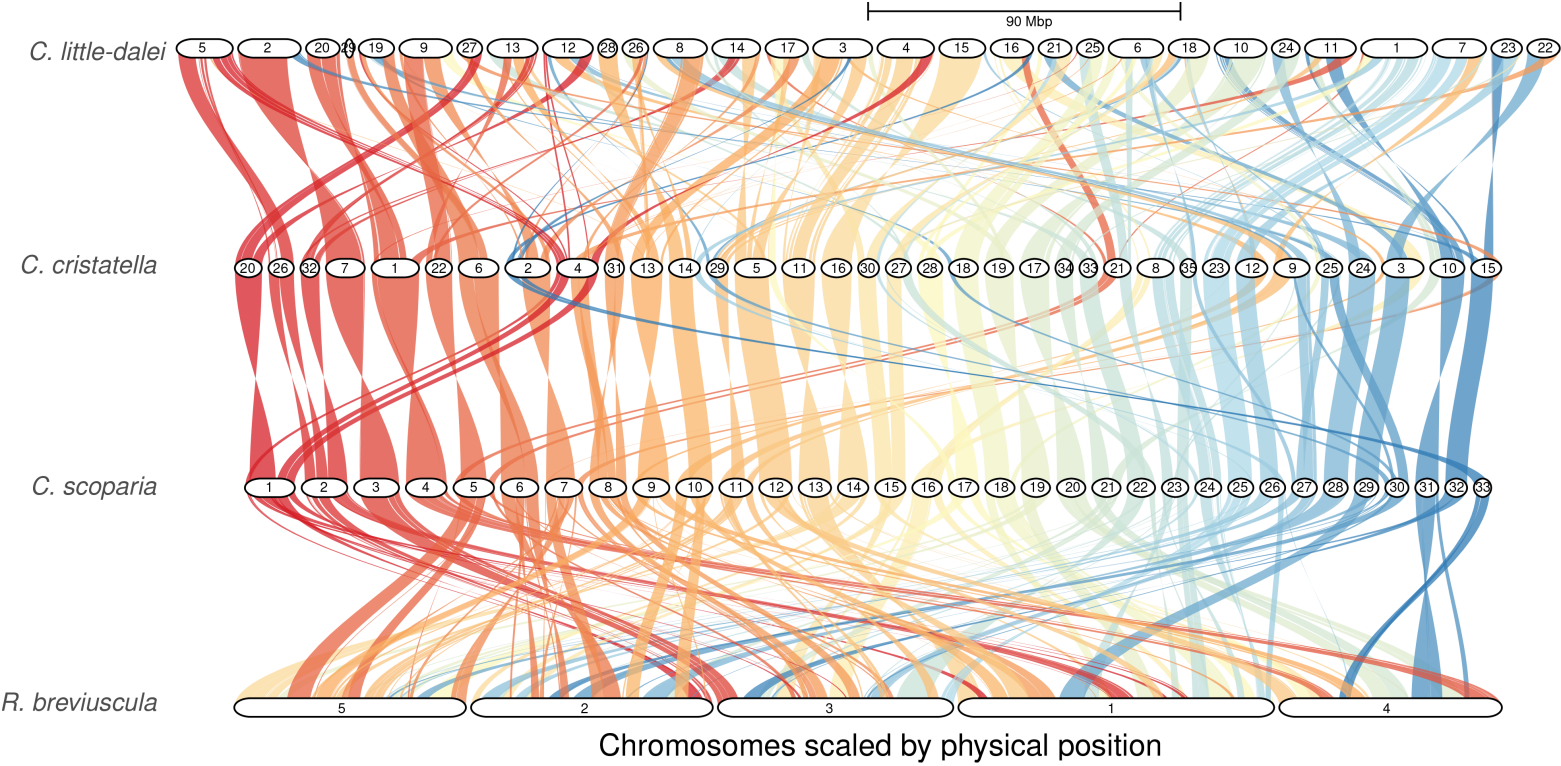
GENESPACE riparian plot obtained using the synteny calculation blocks for *Rhynchopora*, *C. little‐dalei* and *C. scoparia* and *C. cristatella*. Syntenic blocks are colored based on *C. scoparia* chromosomes as a reference.

Synteny between *Carex* and *Rhynchospora* was assessed by reciprocal mapping of the genomes with MUMmer and identification of synteny blocks with SyMAP. This mapping confirms the strikingly high conservation of synteny between *Rhynchospora* and *C. little‐dalei* that has been previously reported (Hofstatter et al., 2022) and further demonstrates that most *C. little‐dalei* chromosomes are entirely syntenic to *R. breviuscula* intrachromosomal regions (Figures 3 and S2). Despite this conserved macrosynteny, 128 rearrangements (fissions, fusions and inversions) were inferred.

The comparison between *Carex* species showed greater synteny. *C. scoparia* and *C. cristatella* genomes are highly collinear and syntenic to each other, with most chromosomes showing no evidence of major rearrangements. Chromosome increase in *C. cristatella* can be mostly explained by fission of relatively few *C. scoparia* chromosomes, for instance from CsChr8 to CcChr4 and CcChr31, and from CsChr15 to CcChr27 and CcChr30. This has possibly increased the chromosome number in the ancestor of *C. cristatella* to *n* = 36, followed by a further reduction by fusion found in CcChr8 (CsChr16 and CsChr24) to reduce it to *n* = 35. In contrast, *C. little‐dalei* showed a more rearranged karyotype, likely due to its more distant relationship between the other two *Carex* species. 128 rearrangements between *Rhynchospora* and *C. little‐dalei* (Figures 3 and S2), 111 rearrangements between *C. scoparia* and *C. little‐dalei* (Figures 3 and S2), 92 rearrangements between *C. cristatella* and *C. little‐dalei* (Figures 3 and S2), and 39 rearrangements between *C. cristatella* and *C. scoparia* (Figures 3 and S2) were estimated in total. The squared number of rearrangements correlates with age of the most recent common ancestor (MRCA) for each pairwise comparison (50 Ma for *Carex* and *Rhynchospora*; 23 Ma for *C. little‐dalei* and the two other *Carex* species; 2 Ma for *C. cristatella* and *C. scoparia*) using a linear model (*r* ^2^ = .892, *p* = .056).

Comparisons of genome assemblies against the linkage map inferred 33 rearrangements between *C. little‐dalei* and *C. scoparia*, 23 between *C. cristatella* and *C. scoparia*, and 14 (plus 4 inversions) between *C. scoparia* populations. ChromEvol modelling reconstructed 277 rearrangements between *C. little‐dalei* and *C. scoparia*, 276 between *C. little‐dalei* and *C. cristatella* and 29 between *C. cristatella* and *C. scoparia*. These estimations of rearrangements are higher for chromEvol when comparing distantly related species, similar for all approaches when comparing closely related species and higher for genome versus linkage group map when comparing populations.

### 3.4 Genomic features of conserved versus rearranged genome areas

To identify genomic features that might be associated with rearrangement hotspots, six categories of genomic repeats were mapped to each *Carex* genome: satellite DNA (satDNA), transposable elements (TEs), Ty3, Ty1‐Copia, class II non‐LTR LINEs, and tRNAs (Figure S3). All features were classified, in a multiple regression model, as falling in a conserved or rearranged area of the genome, and the strength of association with genome rearrangement estimated using generalized linear models. satDNA predicts significantly conserved versus rearranged genomic areas when comparing *C. scoparia* linkage groups against *C. scoparia* and *C. cristatella* genomes (Table S3).

The abundance and distribution of most repeat classes varied, however, along chromosomes in response to the number of rearrangements. Comparing genome assemblies using a window‐based approach, significant interactions were detected between the number of chromosome rearrangements and the distance of each window to the midpoint of the chromosomes for the number of genes, centromeric DNA, Ty3 and Ty1‐Copia for both *C. scoparia* and *C. cristatella* (Table S4) and ClassII TE for *C. cristatella*. Predicted marginal effects suggest that for both species a higher amount of repetitive and centromeric DNA close to the telomeres for chromosomes that have undergone more rearrangements (Figure S4). Conversely, conserved chromosomes have a higher gene density towards the midpoint of the chromosomes (Figure S4).

## 4 DISCUSSION

Our work demonstrates conserved genome synteny between *Carex* species (Figures 2 and 3) despite millions to tens of millions of years since lineage divergence (Martín‐Bravo et al., 2019) and high rates of chromosome evolution (Figure 1; Table S1). It also demonstrates strongly conserved synteny between *Carex* and *Rhynchospora* over an estimated 50 Ma of divergence (Figure 3). The inferences of (i) smaller number of rearrangements than expected between *C. little‐dalei* and distantly related *Carex* species and *Rhynchospora*, (ii) more or less expected number of rearrangements between closely related species (*C. scoparia* and *C. cristatella*), and (iii) higher number of rearrangements than expected within a species (*C. scoparia*) together suggest genomic constraints on rearrangements that act over macroevolutionary timeframes, despite high rates of genome evolution within species. This may be interpreted as evidence of either conserved genomic hotspots of chromosome evolution, where fissions and fusions occur repeatedly in the evolution of sedges; or selection against rearrangements, which only becomes obvious at macroevolutionary scales.

Our results also suggest heterogeneity in the rates of chromosome evolution across the phylogeny of genus *Carex*, which is supported by comparative genomics. While we found a high number of rearrangements among closely related species (*C. cristatella* vs. *C. scoparia*, diverged ca. 2 Ma) or within species (*C. scoparia* populations) of the *Cyperoideae* clade, a previous study has demonstrated only two rearrangements between sedge species estimated to have diverged three times as long ago as *C. cristatella* and *C. scoparia* (Ning et al., 2023). While methodological differences between our studies may account for some of this discrepancy, the finding is further supported by the heterogeneity we find across clades in our phylogenetic study of the evolution of chromosome number (Figure 1). Our work points to the need for more comparative genomic studies to document and explain variation in genome evolution rates in holocentric clades.

Rearrangement hotspots in plant genomes are often associated with repetitive DNA (Raskina et al., 2008; Sutherland et al., 1998), including amplified telomeric repeats or microsatellite repeats (Grabowska‐Joachimiak et al., 2015), and ribosomal DNA (Huang et al., 2008, 2009; Lan et al., 2016; Raskina et al., 2008). Such rearrangement hotspots are also common in different lineages of animals (Ruiz‐Herrera et al., 2002; Toledo et al., 2000), including humans (Hellman et al., 2002; Kaufmann & Reiss, 1999). Yet holocentric lineages, where rearrangements are particularly common, are relatively uncharacterized in this regard. Evidence for rearrangement hotspots in holocentric organisms was previously restricted to the peach‐potato aphid (*Myzus persicae*; Manicardi et al., 2015) and possibly the silkworm (*Bombyx mori*) and relatives (d'Alençon et al., 2010). Conservation of synteny blocks might, in fact, be due to conserved patterns of chromosome fusion across Lepidoptera, where two species in separate genera have undergone parallel chromosomal fusions involving the same six ancestral chromosomes (Ahola et al., 2014) and rearrangements in at least one genus are associated with repeat DNA (Höök et al., 2023). In the sedge genus *Rhynchospora*, end‐to‐end chromosome fusions may be frequently associated with centromeric repeats and, at lower frequency, ribosomal and telomeric DNA (Hofstatter et al., 2022). These findings and ours lend credence to pre‐genomic hypotheses that hotspots for chromosome rearrangements might explain the rapid fixation of new cytotypes in natural populations of *Carex* (Luceño, 1994; Roalson, 2008; Whitkus, 1988), a genus renowned for exceptionally long intraspecific diploid chromosome number series (Escudero et al., 2008; Escudero, Maguilla, & Luceño, 2013; Escudero, Weber, & Hipp, 2013).

Recently, it has been shown that holocentric organisms have unusual common features such as uniform distribution of genes, repeats as well as eu‐ and heterochromatin marks along the whole chromosomes, affecting the overall genome architecture (in Homoptera and holocentric nematodes: Mandrioli & Carlo Manicardi, 2012; in Lepidoptera: Haenel et al., 2018; in sedges: Hofstatter et al., 2022). For the beak‐sedge genus *Rhynchospora*, where holocentromeres have been studied in detail, centromeric arrays of repetitive DNA are ca. 20 kb long and intermingled with gene‐coding sequences and TE (Hofstatter et al., 2022; Marques et al., 2015). As observed in *Rhynchospora* (Hofstatter et al., 2022), we hypothesized that regions between synteny blocks should be in the centromeric arrays of repetitive DNA, so chromosome rearrangements would not break up gene arrays. We expected significant differences between conserved versus the rearranged regions, which we predicted to be enriched in genes or repetitive sequences, respectively. Our modelling of genome evolution points out the presence of hotspots of fusion and fission and we have been able to detect differences in the underlying genomic features between conserved and rearranged genome regions. Specifically, repeat DNA seems to be related to these hotspots for chromosome rearrangements.

The presence of hotspots for fission and fusion may have important consequences for chromosomal speciation in holocentric species. One of the most important critiques to the model of hybrid dysfunction of chromosomal speciation is that the evolution of individuals with strongly underdominant karyotypes is needed to drive speciation, yet strong selection gives such evolutionary novelties no one to mate with (Husband, 2000).The mechanisms for establishment of such underdominant, speciation‐driving karyotypes might include genetic drift, inbreeding, higher fitness of individuals homozygous for the new variant, or meiotic drive (White, 1978). The nature of holocentric chromosomes has already been proposed to facilitate the successful establishment of new karyotypes (Lucek et al., 2022) via the frequent co‐occurrence of different chromosome numbers within species or even within populations. Hotspots of chromosome fusion and fission may also increase fixation of new karyotypes by increasing the probability of convergent genome rearrangements. Previous work demonstrates that chromosome homology cannot be taken for granted in sedges: meiotic complications in interpopulation F1s of *C. scoparia*, for example, reveal different karyotypes between parents that have the same chromosome number (Escudero, Hahn, et al., 2016). Our inference of hotspots for rearrangements may contribute to maintaining species coherence in this species with extraordinary chromosome number and karyotypic variation (Escudero, Weber, & Hipp, 2013), yet strong genomic cohesion (2n = 56 to 70; Escudero et al., 2014; Hipp et al., 2010).

A clinal speciation process for holocentric butterflies and sedges with chromosome variation has been also suggested, as neighbour populations with relatively low differences in chromosome number are often reproductively compatible while geographically distant and chromosomally divergent populations show reduced interfertility (Escudero, Hahn, et al., 2016; Lukhtanov et al., 2018; Whitkus, 1988). These chromosome rearrangements can also entail suppression of recombination when crossing with different cytotypes, which could promote selection of locally adapted genes (Butlin, 2005; Faria & Navarro, 2010). This model of recombination suppression across the length of a chromosome inversion that contains a supergene has experimental support in animals (e.g. Durmaz et al., 2018) including humans (Campoy et al., 2022), holocentric organisms (Joron et al., 2011), and plants (i.e. Lowry & Willis, 2010). Interestingly, based on microscopy, it has long been noted that fission and fusion in holocentric chromosomes may result in trivalent chains during mitotic Metaphase I, resulting from the homologies between one large central chromosome and two smaller lateral chromosomes resulting from fission (Wahl, 1940). The result is a disruption of recombination: chiasmata are never formed between genomic regions that are homologous between the central part of the large chromosome and the distal extremes of the two lateral chromosomes (Márquez‐Corro et al., 2019). The association between chromosome rearrangements (14 fissions and fusions and four inversions) and a lack of recombination when comparing the *C. scoparia* linkage groups with a *C. scoparia* genome assembly is congruent with this observation. Our study thus may provide additional support for disruption of recombination as a mechanism by which holocentric fission and fusion shape how selection acts on recombination. Through their effects on recombination (Escudero et al., 2012; Escudero, Maguilla, & Luceño, 2013) as well as hybrid dysfunction (Escudero, Hahn, et al., 2016), the constraints we document on holocentric chromosome fission and fusion may shape the diversification of holocentric lineages and thus the composition of global biodiversity.

### 4.1 Final remarks

Because holocentric chromosomes have multiple attachment points for microtubules, chromosome fragments can still segregate effectively during meiosis, which reduces the cost of chromosome fissions and fusions (Hipp et al., 2009). Our study demonstrates how synteny can persist in the face of extensive chromosome rearrangements in holocentric species. This suggests the interplay between selection for conserved synteny blocks (potentially similar to supergenes) and large‐scale synteny at macroevolutionary scale on the one hand and chromosome rearrangements facilitating local adaptation at microevolutionary scale on the other hand. This tension is congruent with the fact that the experimental support for chromosomal local adaptation in sedges is stronger at a microevolutionary scale (Escudero, Maguilla, & Luceño, 2013; Márquez‐Corro et al., 2023) than at a macroevolutionary scale (Escudero et al., 2012; Márquez‐Corro et al., 2021; Spalink et al., 2018).

## AUTHOR CONTRIBUTIONS

AH and ME conceived the idea. AM and ME gathered the data. AM, KL and ME performed the analyses. ME drafted a first version of the manuscript reviewed by AH, AM and KL.

## CONFLICT OF INTEREST STATEMENT

The authors have no conflict of interest to declare.

## BENEFIT‐SHARING STATEMENT

Benefits Generated: This is an international research collaboration that did not involve sample collection as genetic data was gathered from previous publications.

## Supporting information


Figure S1.

Figure S2.

Figure S3.

Figure S4.

Table S1.

Table S2.

Table S3.

Table S4.

## Data Availability

Raw sequence reads are deposited in the SRA (BioProjects PRJNA723756 and PRJNA784789 and GenBank accessions CM022079–CM022107). Linkage mapping data are deposited here https://zenodo.org/record/1196852. *Carex little‐dalei* https://doi.org/10.5281/zenodo.8138413. *Carex cristatella* https://doi.org/10.5281/zenodo.8138415. *Carex scoparia* https://doi.org/10.5281/zenodo.8138417.
