# Supplementary figures and images for: Genomic hotspots of chromosome rearrangements explain conserved synteny despite high rates of chromosome evolution in a holocentric lineage
Source: Mol Ecol. 2023 Jul 24;33(24):e17086. doi: 10.1111/mec.17086 (PMC11628656; doi:10.1111/mec.17086)

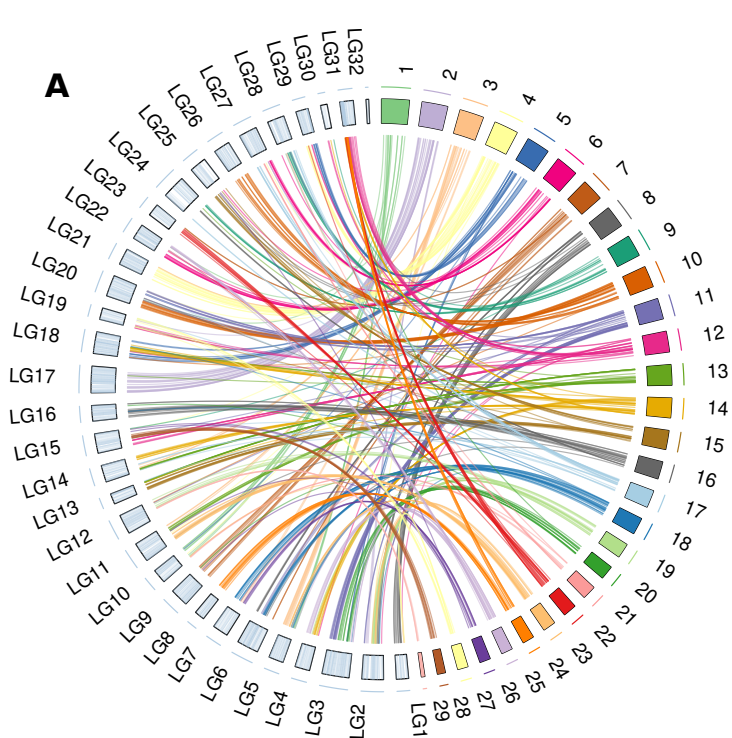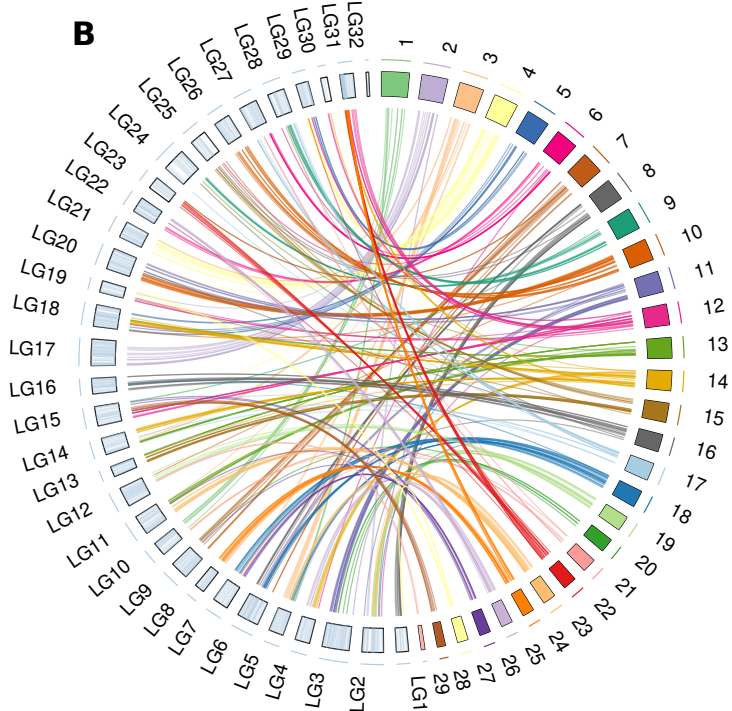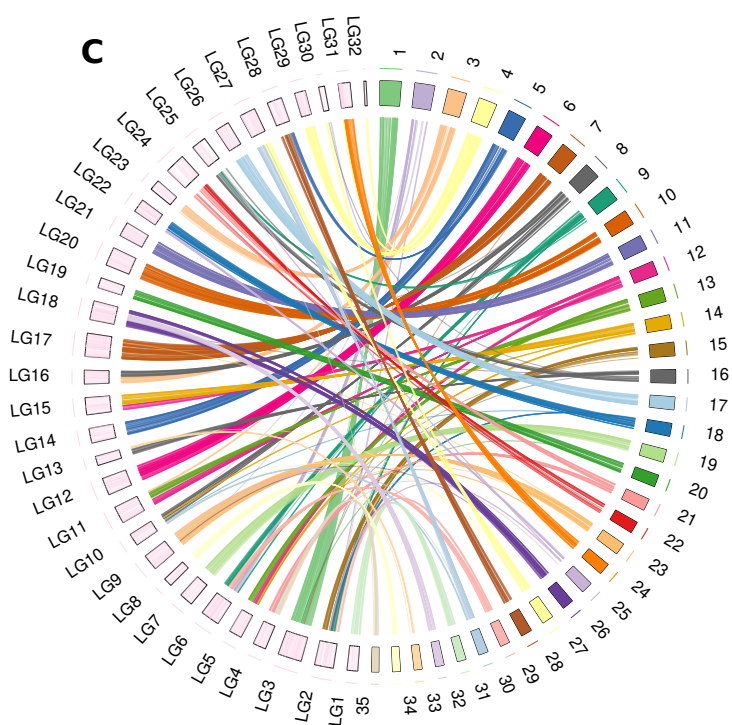

Supplement: Supplementary file 1 — Figure S1. [file MEC-33-e17086-s008.pdf]

Carex littledalei synteny to Rhynchospora breviuscula

A

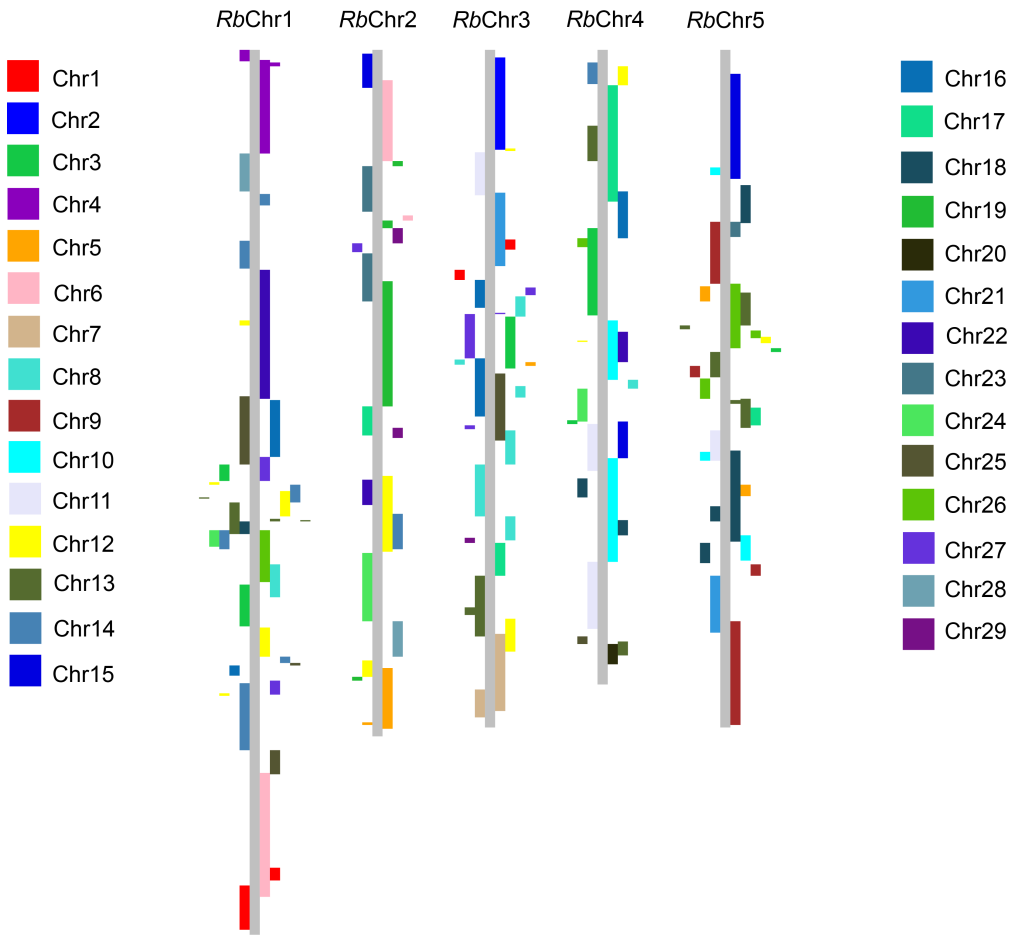

B

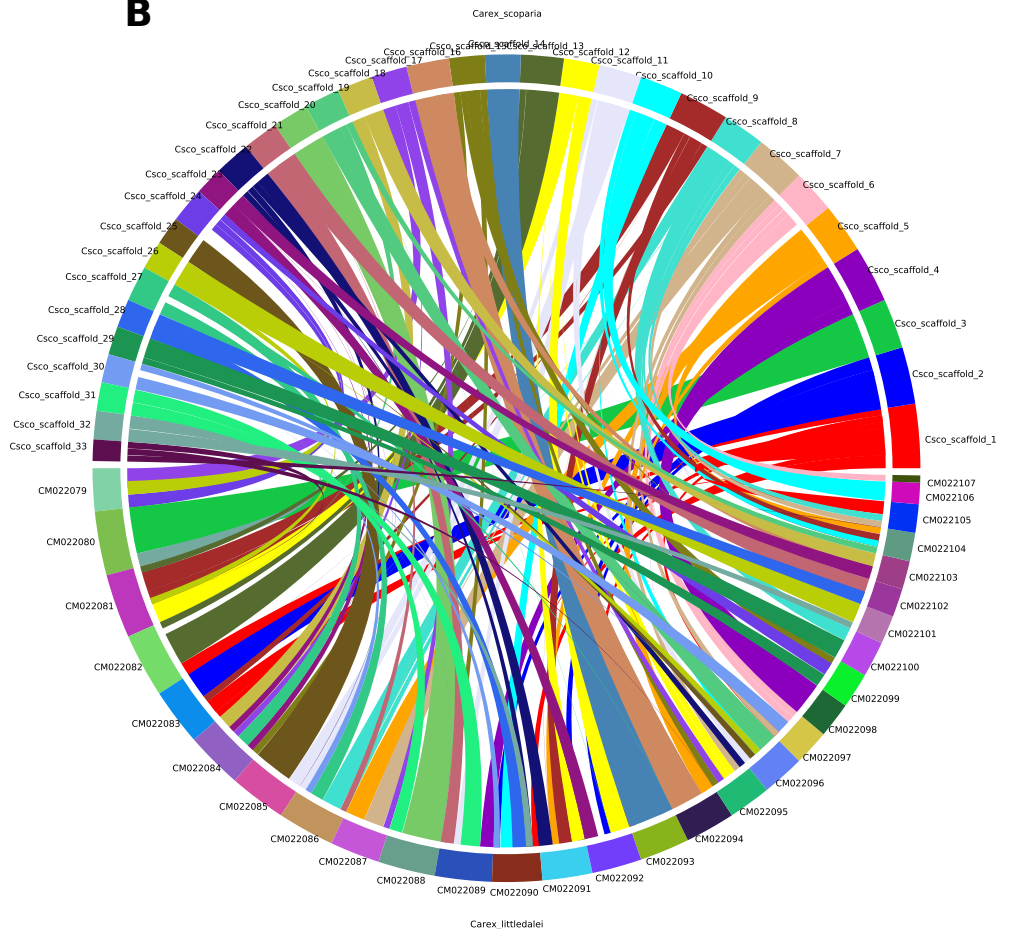

C

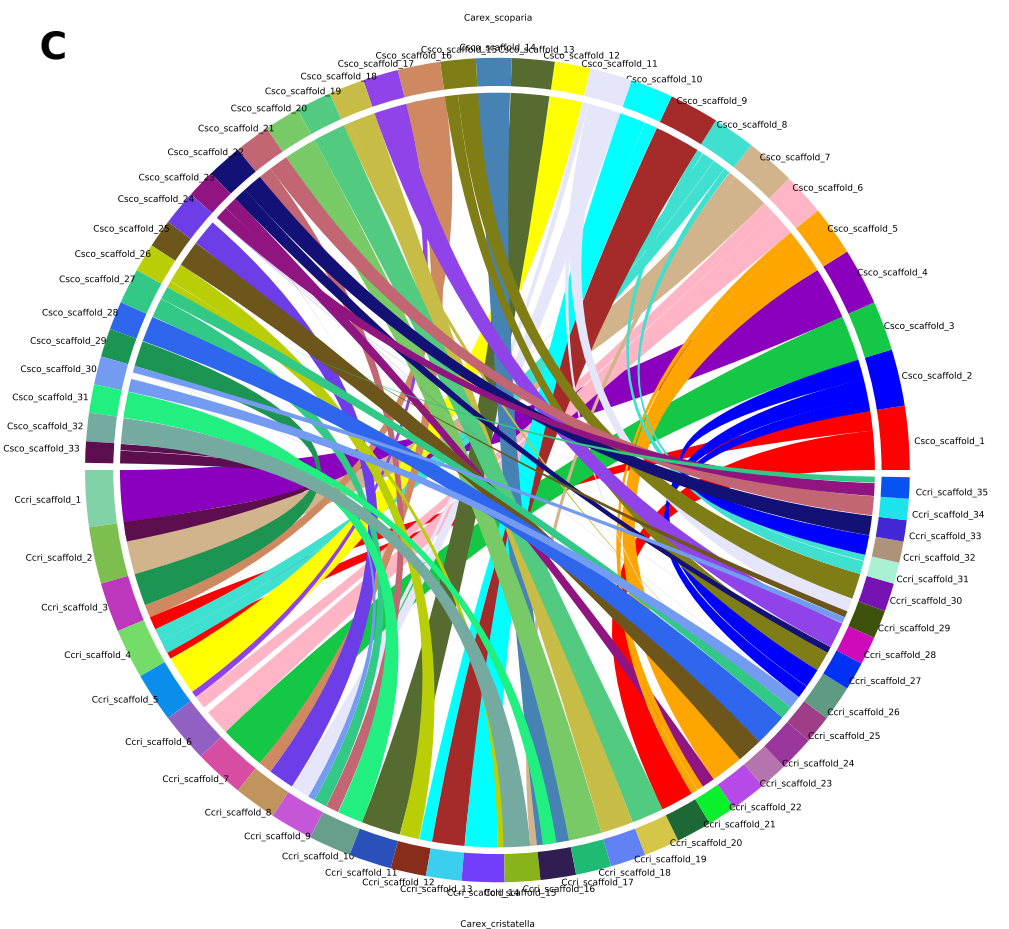

D

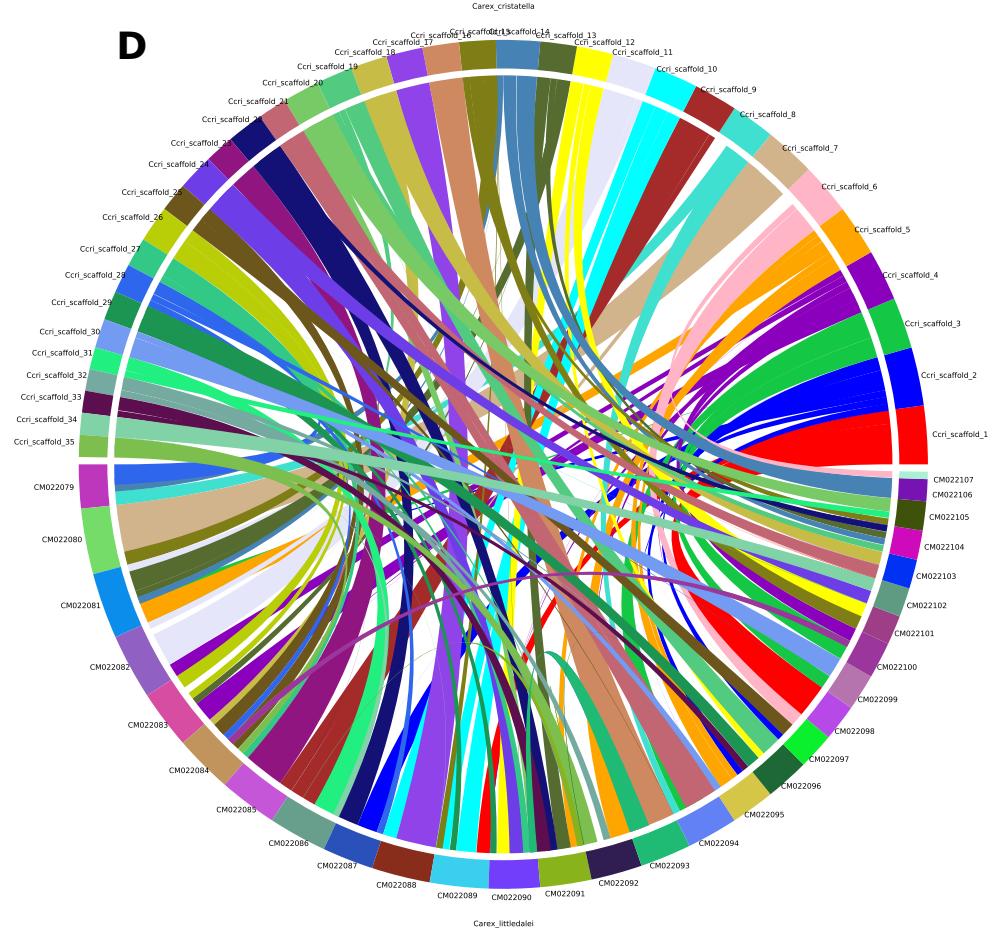

Supplement: Supplementary file 2 — Figure S2. [file MEC-33-e17086-s007.pdf]

**A**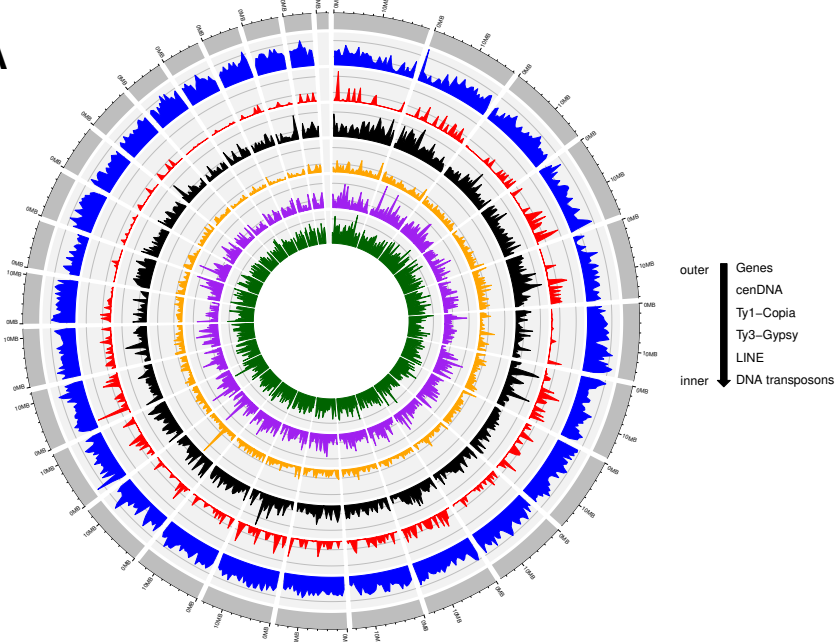**B**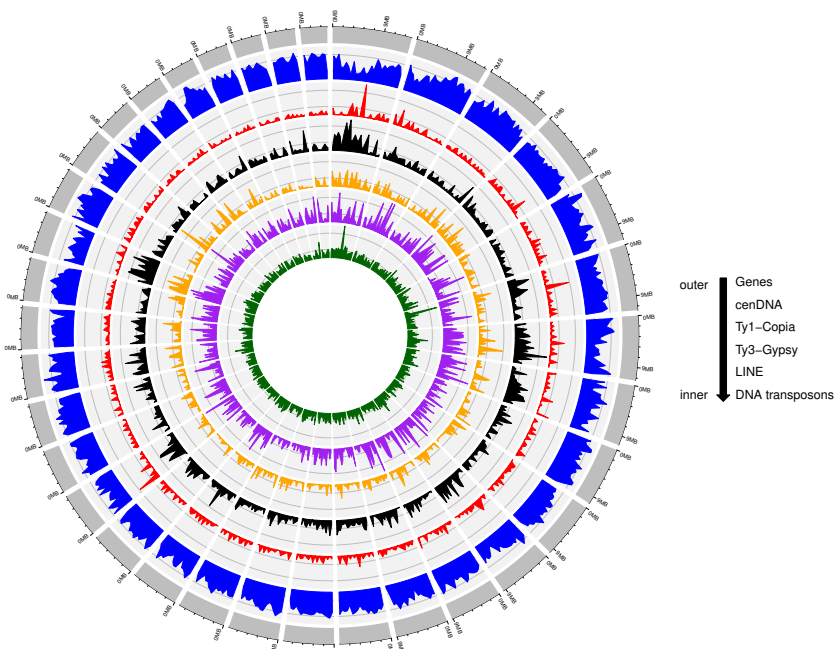**C**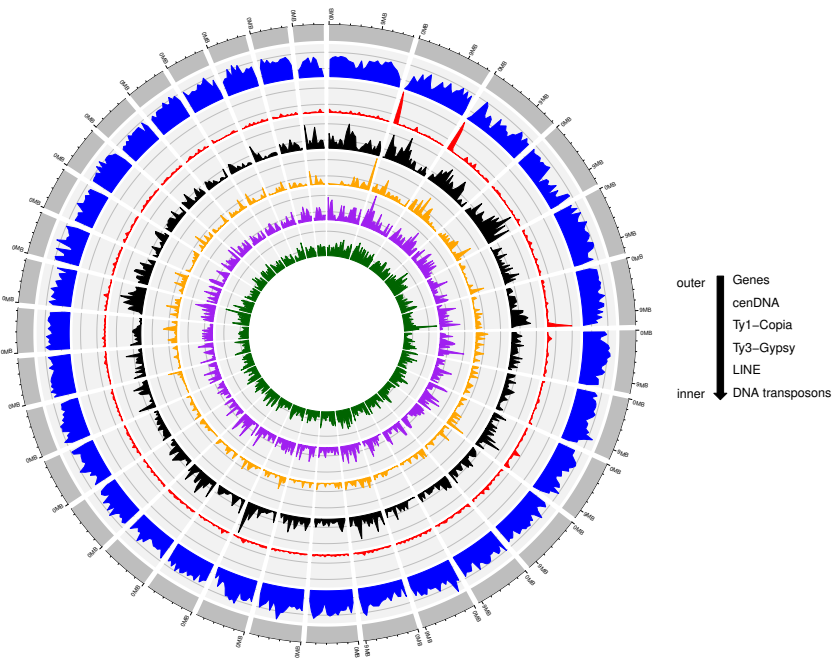

Supplement: Supplementary file 3 — Figure S3. [file MEC-33-e17086-s006.pdf]

*C. cristatella*

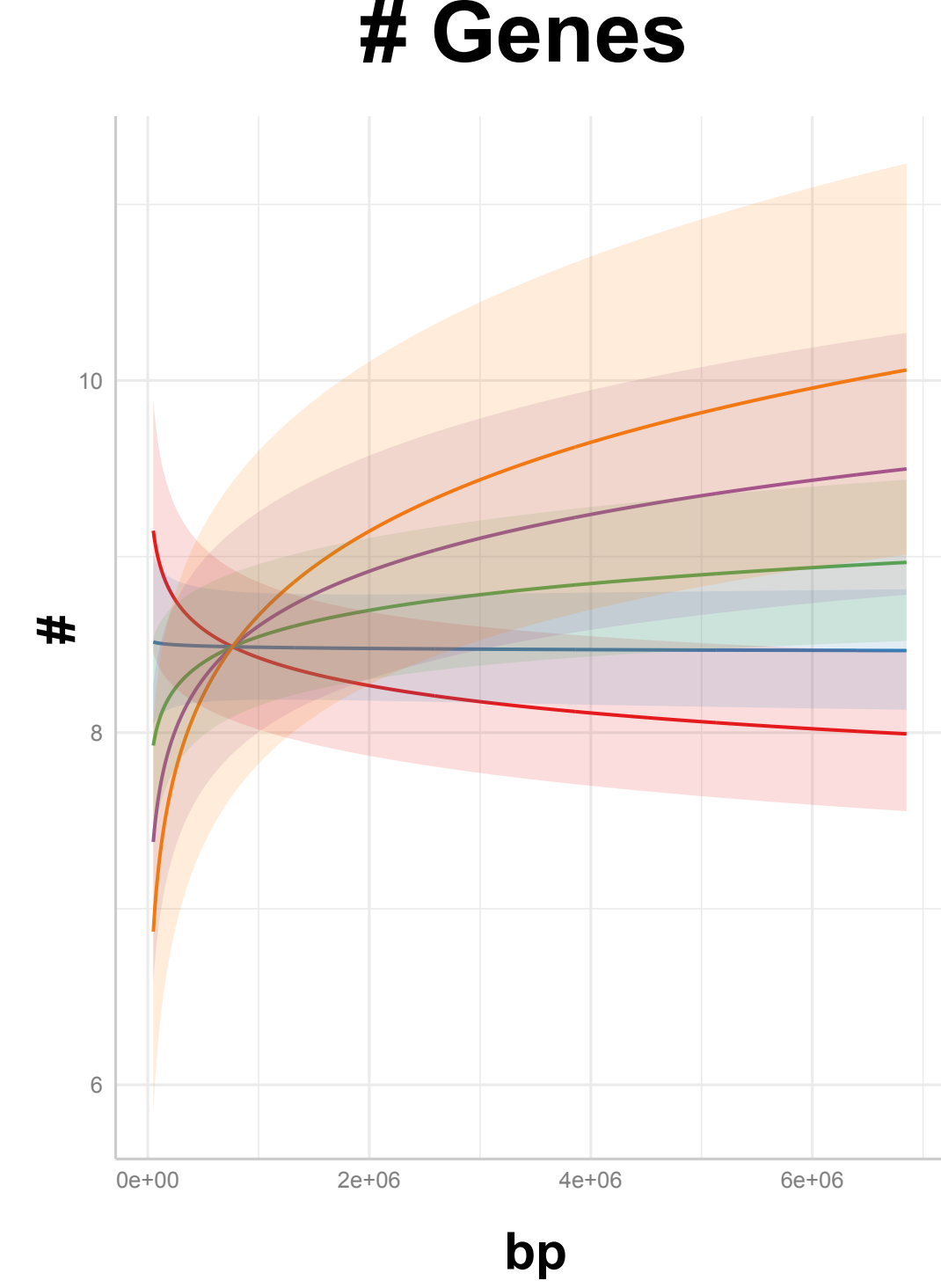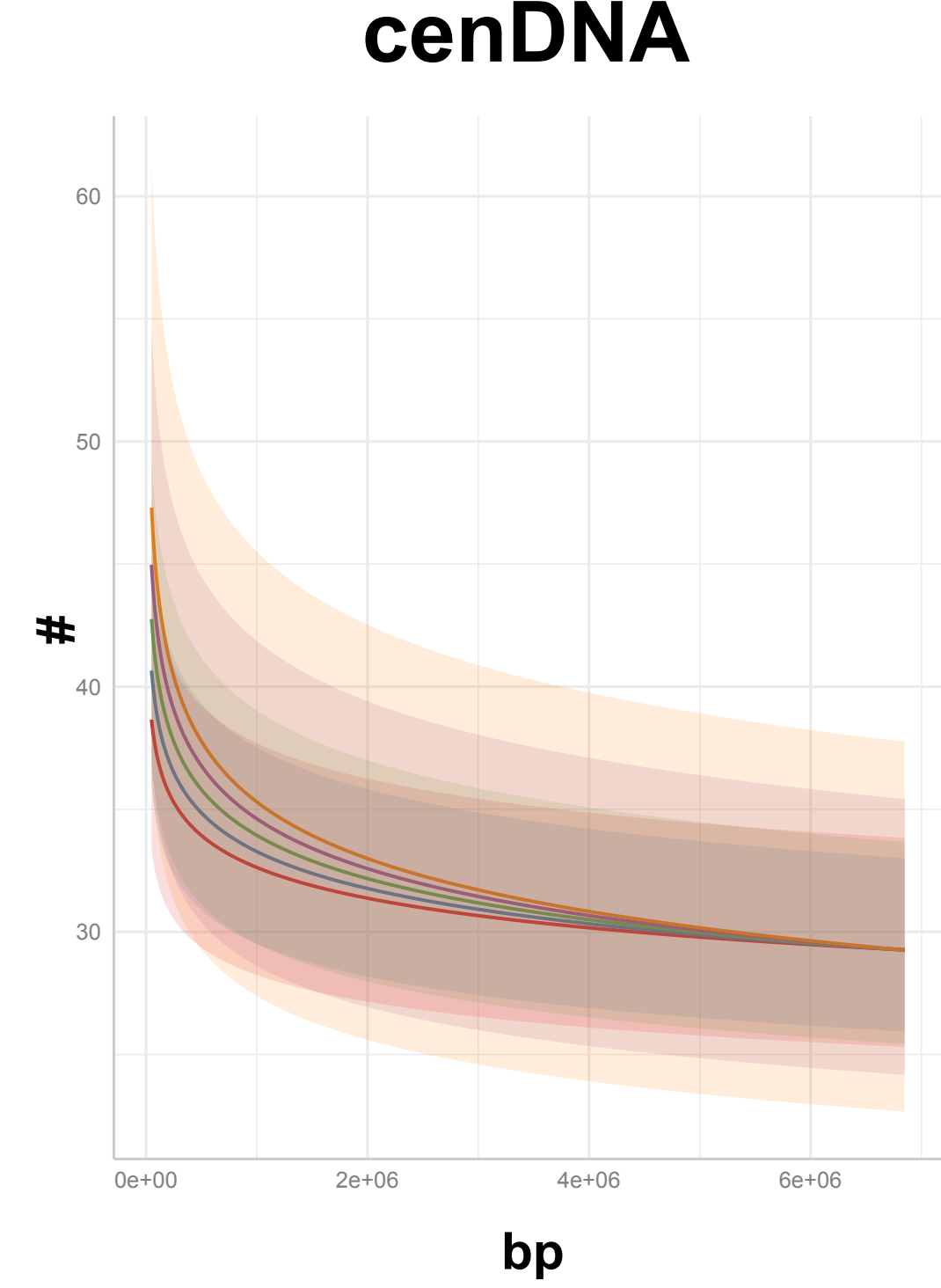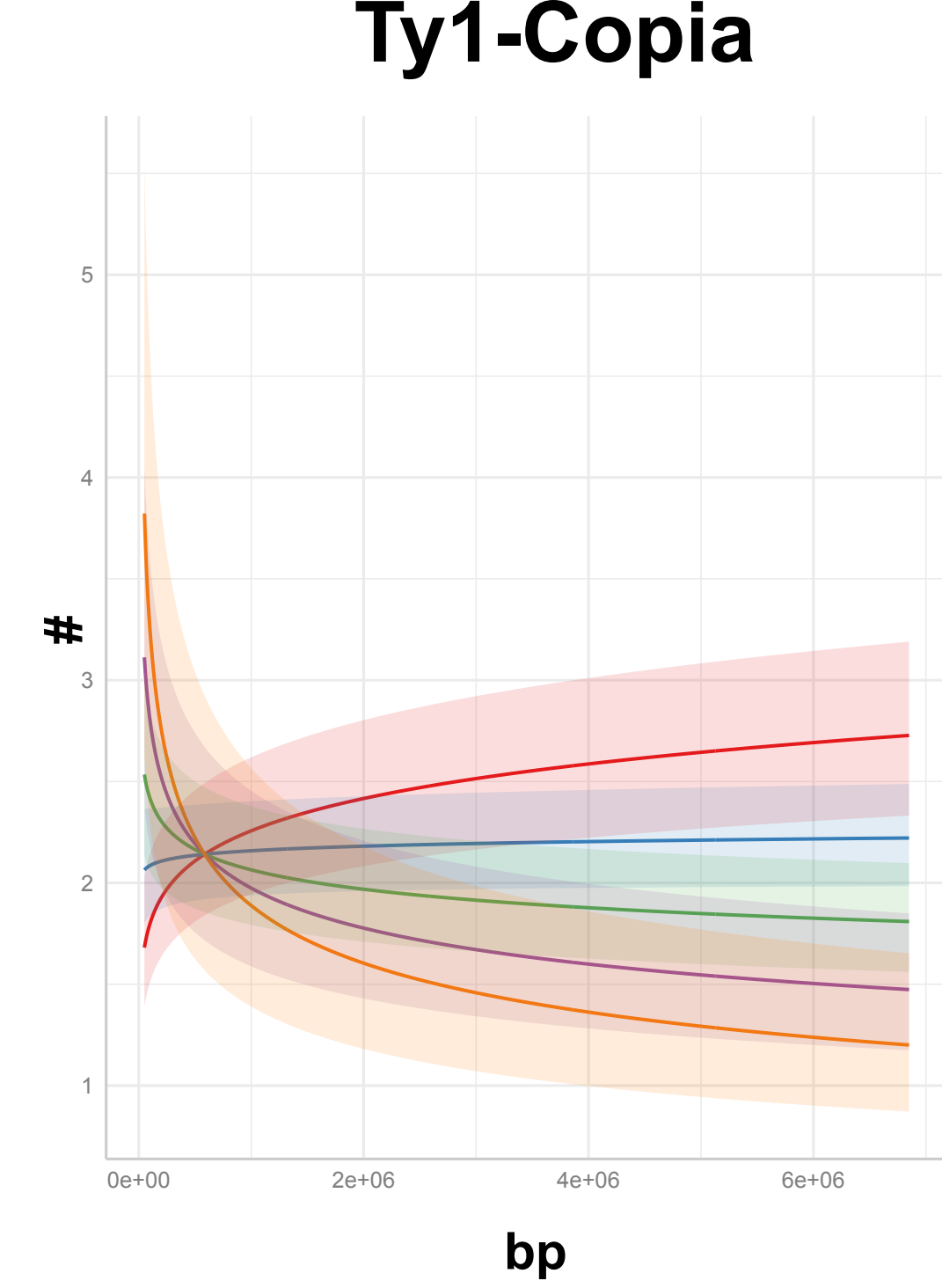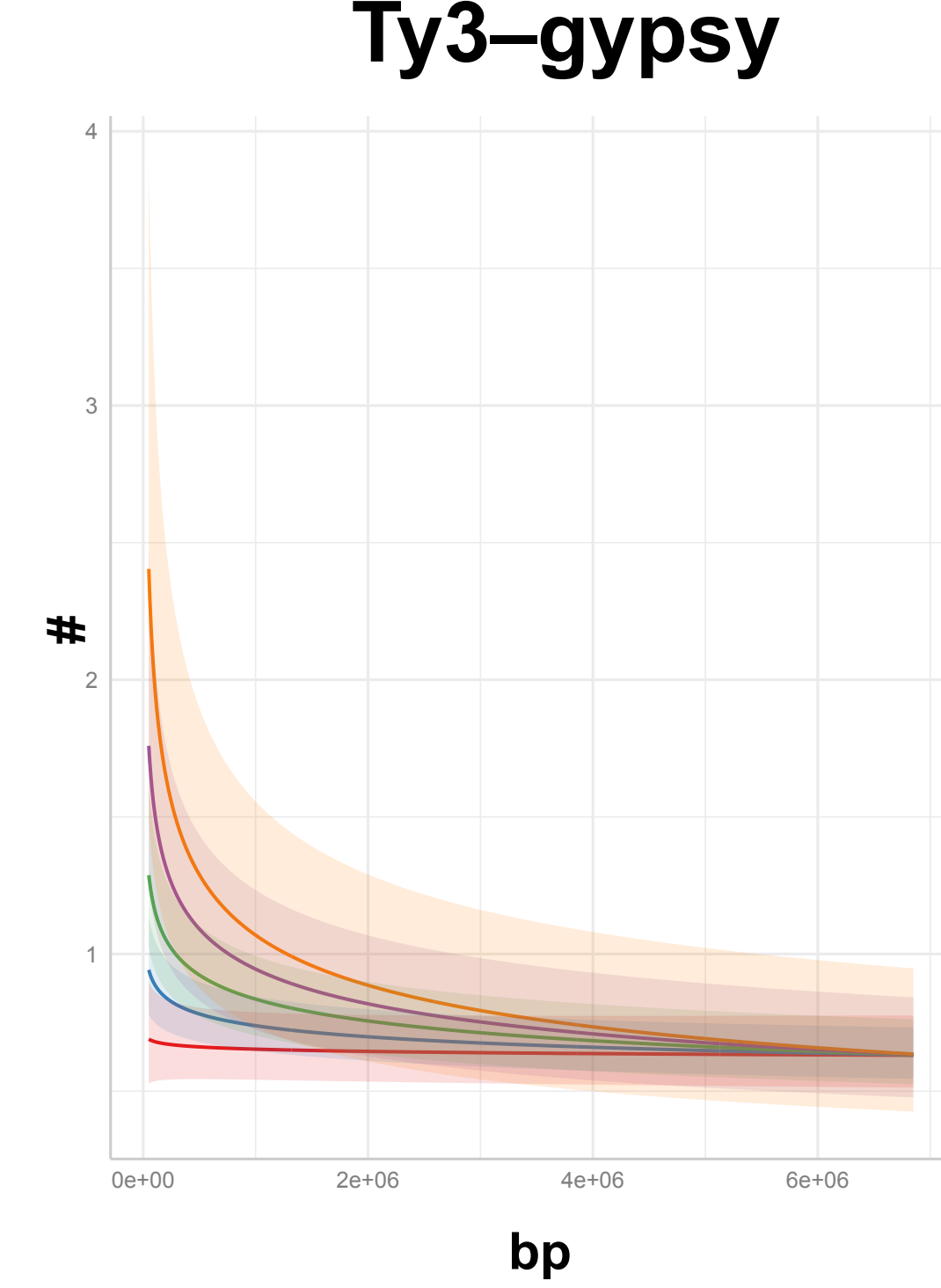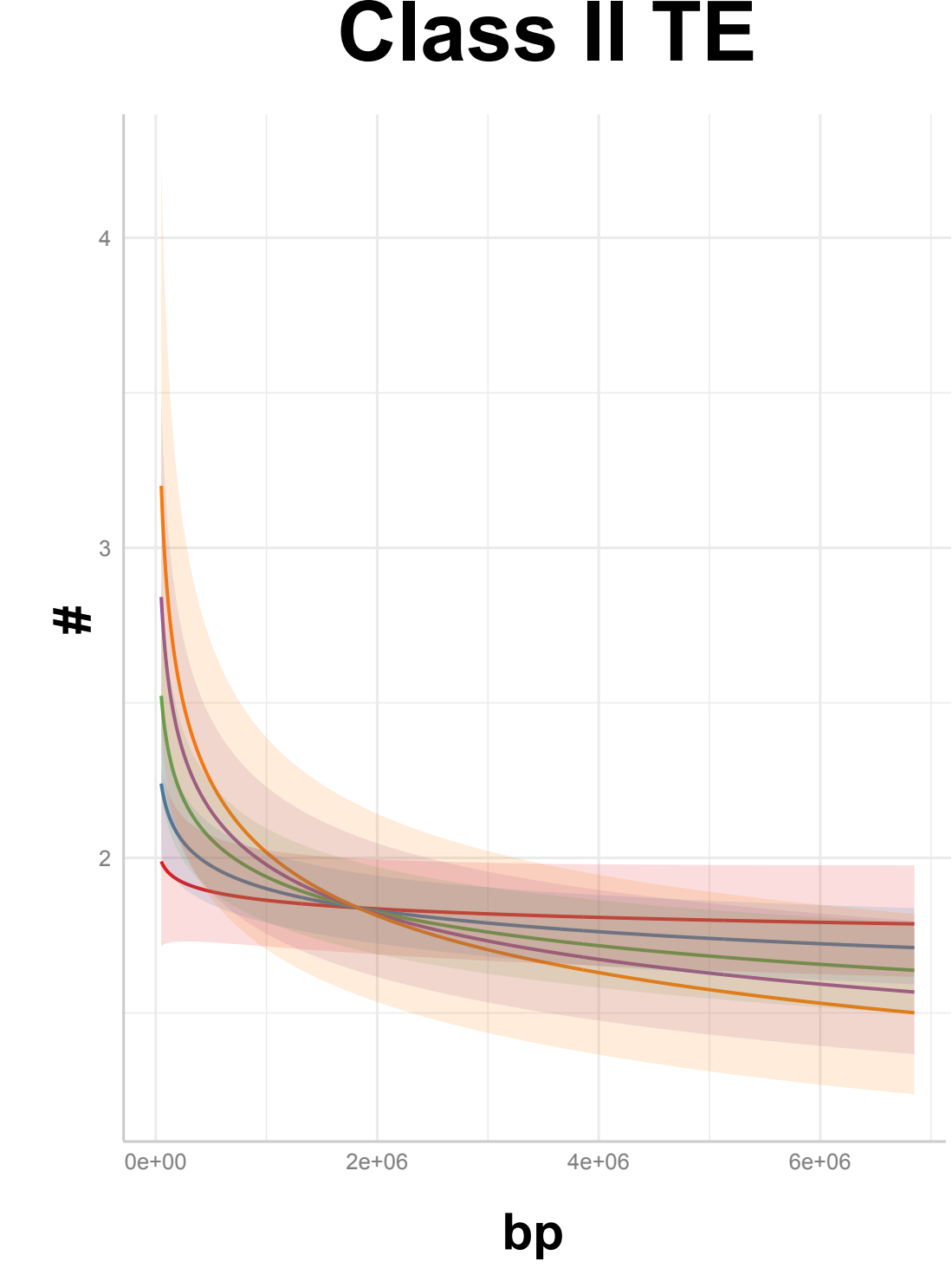

*C. scoparia*

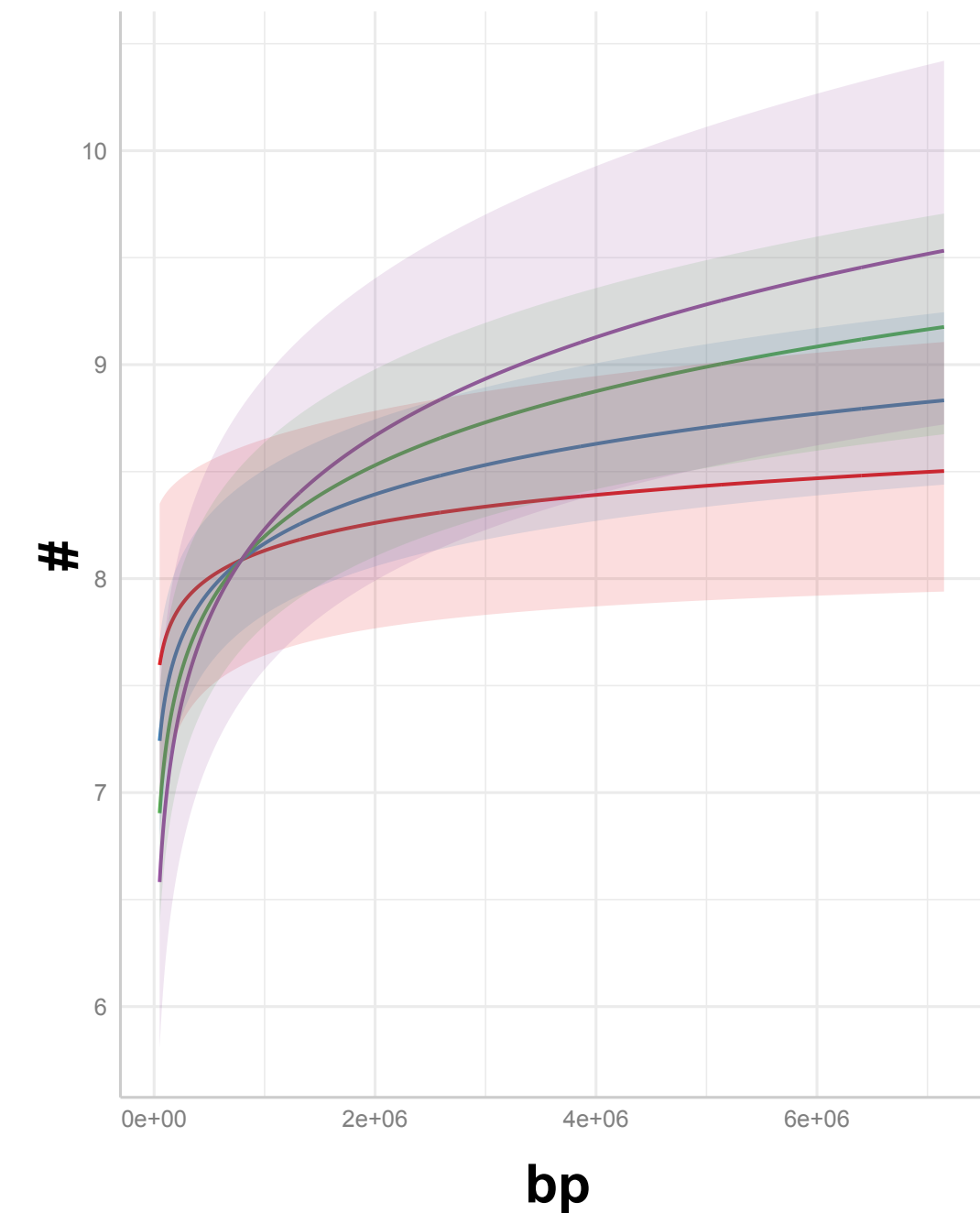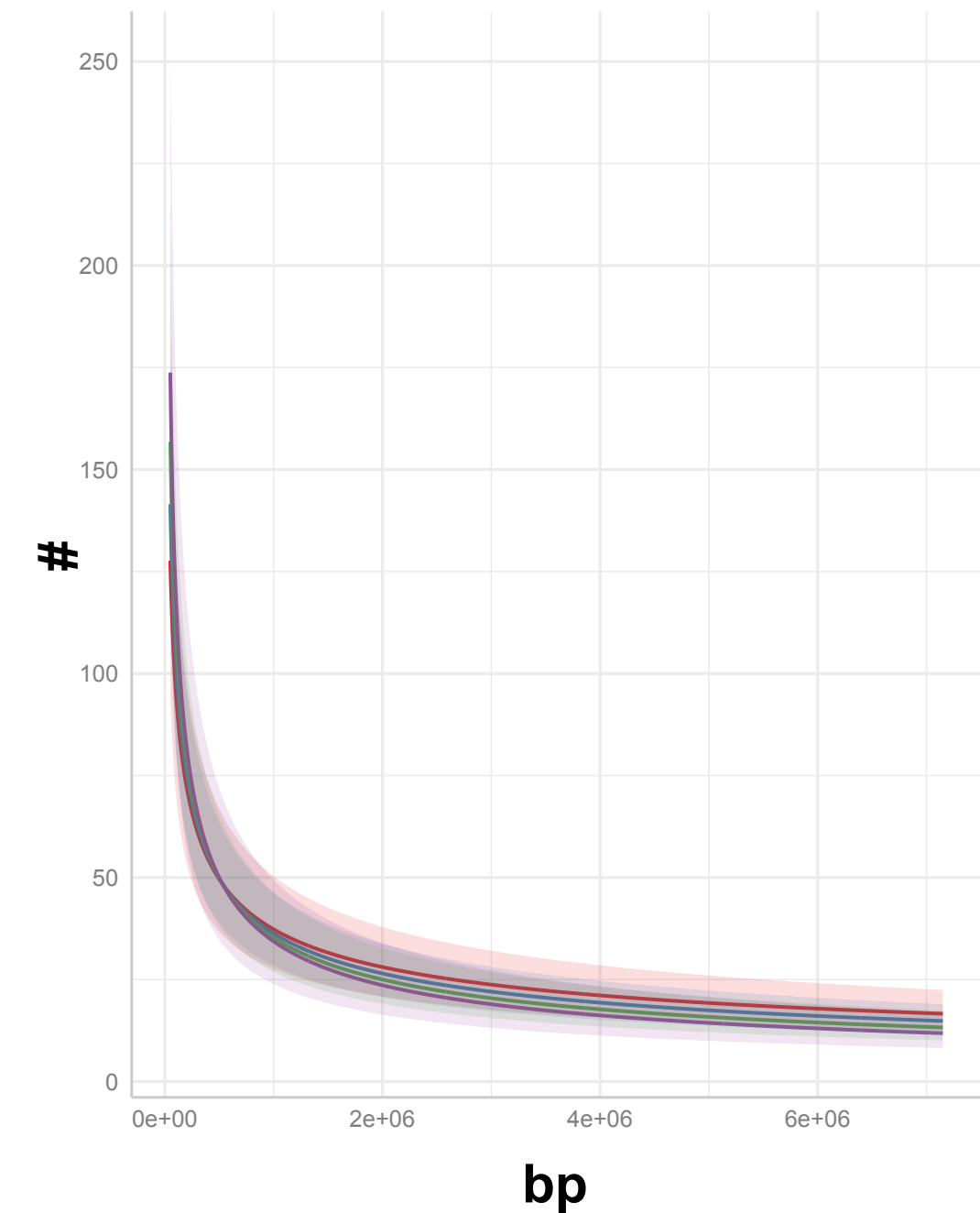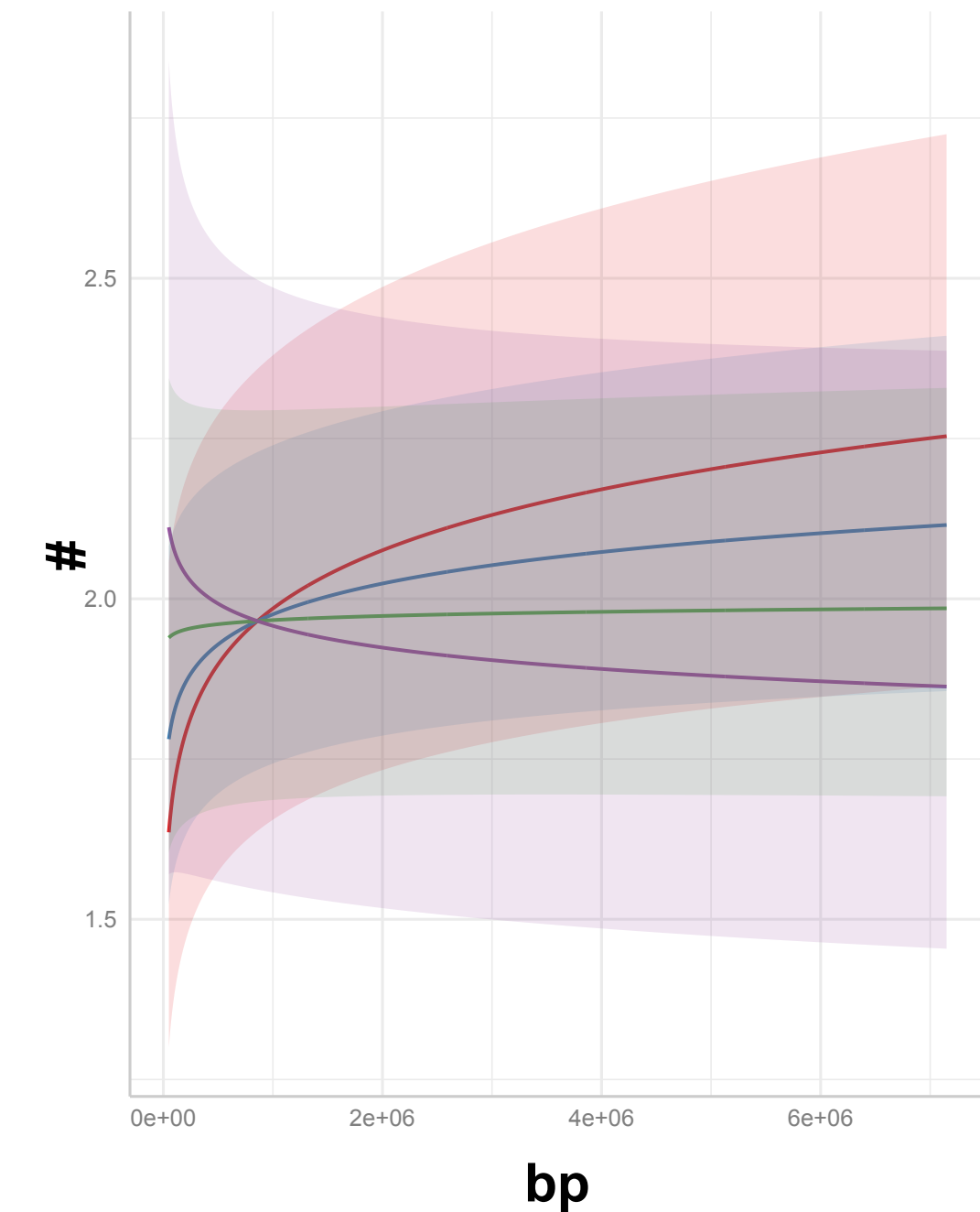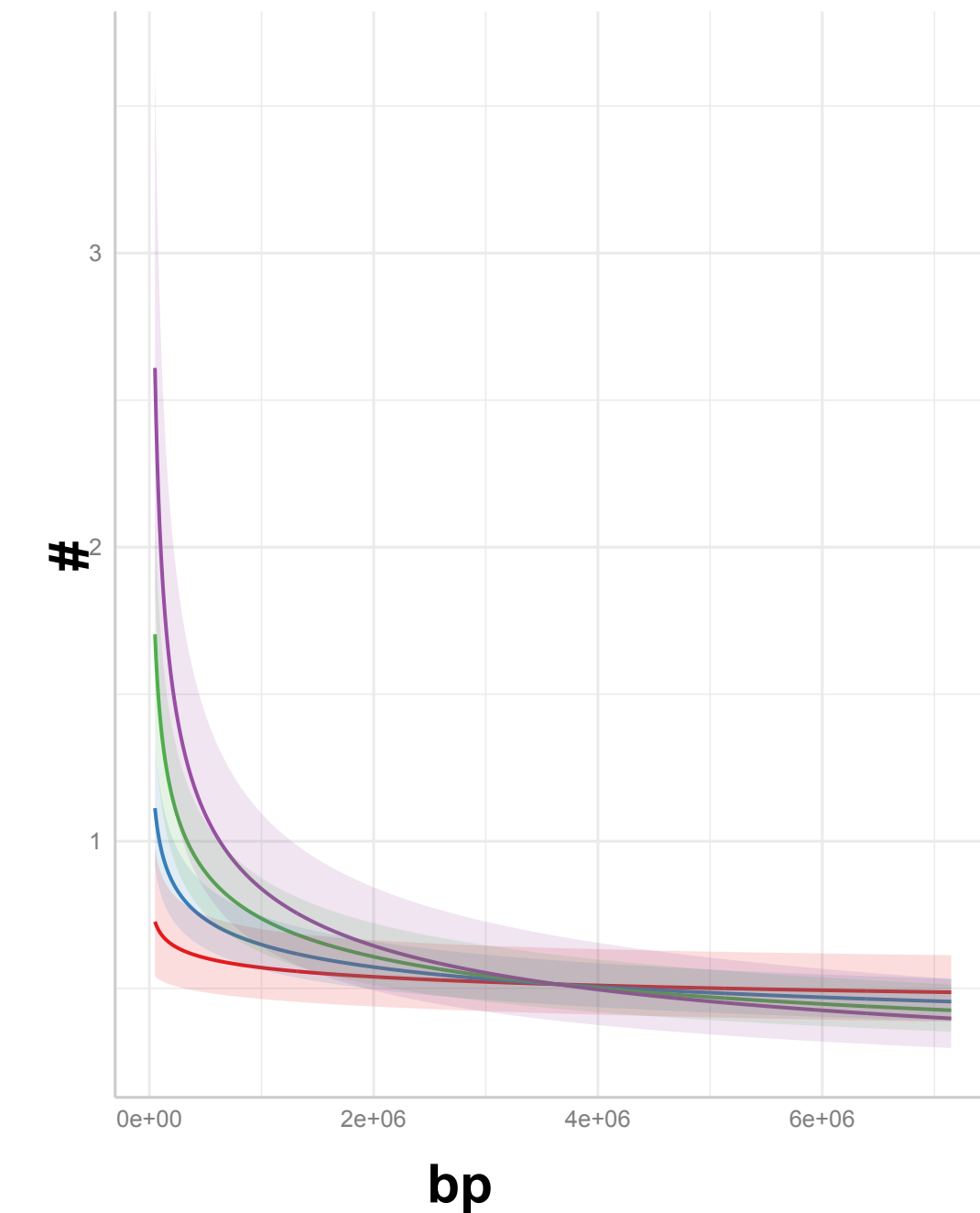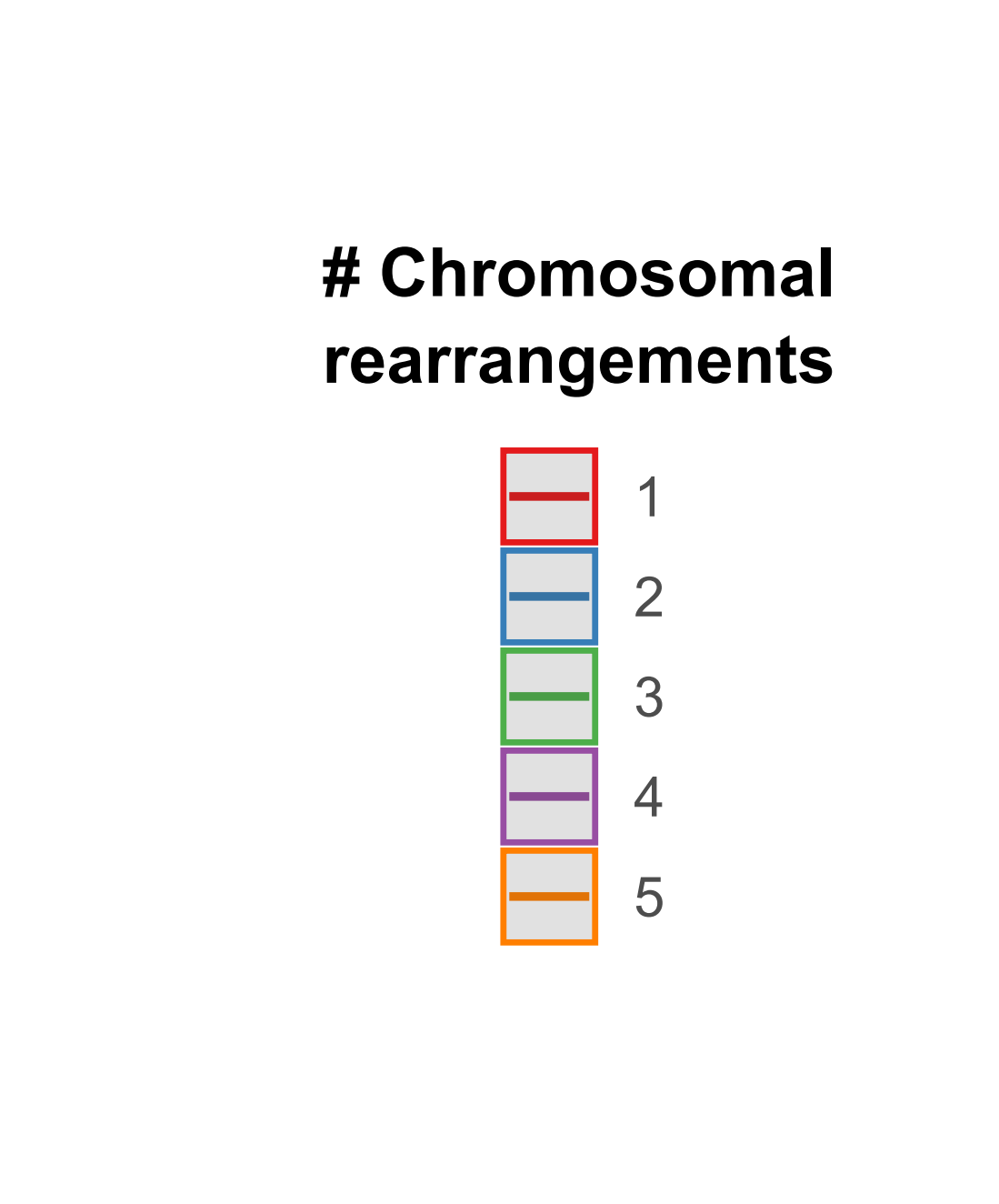

Supplement: Supplementary file 4 — Figure S4. [file MEC-33-e17086-s003.pdf]
