# Supplementary material for: Genomic hotspots of chromosome rearrangements explain conserved synteny despite high rates of chromosome evolution in a holocentric lineage
Source: Mol Ecol. 2023 Jul 24;33(24):e17086. doi: 10.1111/mec.17086 (PMC11628656; doi:10.1111/mec.17086)
Supplement: Supplementary file 5 — Table S1. [file MEC-33-e17086-s005.docx]

**Supplementary Table 1**. Results from BAMM analyses. The rates of evolution (lam1) and the growth/decay parameter (lam2) are indicated for the background (node 756) and 124 inferred shifts (rest of the nodes). The time from the root (0 my) to the present are indicated for each node.

| node | time | lam1 | lam2 |
| --- | --- | --- | --- |
| 756 | 0 | 42.0722 | 0.0835646 |
| 760 | 11.2208 | 1.98538 | 0.0121732 |
| 809 | 18.1865 | 0.915956 | -0.00093534 |
| 53 | 19.6948 | 0.794229 | 0.0120326 |
| 806 | 22.3643 | 6.82192 | 0.0219551 |
| 1494 | 22.9706 | 1.15626 | 0.0325631 |
| 1 | 24.88905 | 0.314347 | 0.02386877 |
| 768 | 25.527 | 0.71604 | 0.0664785 |
| 1490 | 26.1164 | 1.06969 | 0.0552612 |
| 1411 | 26.5562 | 1.65271 | -0.00742176 |
| 770 | 27.4925 | 0.322841 | -0.0741955 |
| 849 | 27.8286 | 1.44928 | 0.0662482 |
| 1013 | 27.9849 | 0.928253 | -0.0217347 |
| 1423 | 28.3512 | 0.476472 | 0.0241142 |
| 28 | 28.50465 | 0.469205 | -0.0491765 |
| 875 | 28.8853 | 2.604 | -0.0692693 |
| 1237 | 29.1017 | 0.914363 | 0.118202 |
| 301 | 29.17595 | 0.724992 | -0.063234 |
| 8 | 29.1847 | 0.885564 | -0.0394906 |
| 1413 | 29.2675 | 2.20058 | 0.0964557 |
| 1401 | 29.388 | 0.182438 | 0.0605669 |
| 43 | 29.5801 | 2.32465 | 0.000978338 |
| 554 | 29.6026 | 2.17148 | 0.019897 |
| 745 | 29.6028 | 3.86382 | 0.0886299 |
| 1016 | 29.6886 | 0.274514 | -0.052813 |
| 650 | 29.7003 | 3.15376 | -0.025963 |
| 300 | 29.7136 | 2.46444 | 0.00443626 |
| 51 | 29.7382 | 0.847692 | -0.00406983 |
| 54 | 29.8262 | 0.865731 | -0.0276052 |
| 1138 | 29.9726 | 1.72894 | 0.00474865 |
| 52 | 30.0153 | 0.945692666666667 | -0.004150733333333 |
| 1068 | 30.0616 | 1.65106 | -0.0442138 |
| 86 | 30.0866 | 0.0140355 | -0.0595126 |
| 293 | 30.1807 | 0.434717 | 0.0102155 |
| 1018 | 30.3937 | 0.629528 | -0.0445543 |
| 161 | 30.6949 | 0.56483 | 0.0326038 |
| 873 | 30.7028 | 1.46438 | 0.0331555 |
| 751 | 30.7529 | 0.485741 | -0.0443062 |
| 1241 | 30.7622 | 1.36319 | -0.00635493 |
| 247 | 30.7966 | 1.24456 | 0.0315416 |
| 612 | 30.819 | 1.01132 | -0.0177873 |
| 1381 | 30.8887 | 1.4972 | 0.0847485 |
| 55 | 30.9735 | 3.92722 | 0.069442 |
| 644 | 31.00995 | 0.4538595 | -0.00768635 |
| 32 | 31.0283 | 0.128124 | 0.00886461 |
| 1170 | 31.126 | 1.30601 | 0.0400384 |
| 663 | 31.1363 | 0.214005 | 0.0173353 |
| 798 | 31.1368 | 1.06917 | -0.000902912 |
| 555 | 31.221 | 0.0804886 | -0.0453995 |
| 395 | 31.2316 | 1.46995 | 0.0178582 |
| 1198 | 31.2971 | 0.777204 | -0.0909833 |
| 904 | 31.2996 | 0.998452 | -0.0850742 |
| 1156 | 31.3882 | 0.605496 | 0.0443012 |
| 845 | 31.4158 | 1.85979 | -0.0107506 |
| 45 | 31.418 | 0.123239 | -0.0411075 |
| 755 | 31.6032 | 1.2398225 | -0.01377975 |
| 846 | 31.6048 | 1.87133 | -0.0998986 |
| 605 | 31.624 | 0.826985 | 0.0299915 |
| 16 | 31.6562 | 0.27754 | -0.0545119 |
| 538 | 31.7475 | 0.137952 | 0.0394666 |
| 1210 | 31.7744 | 4.74406 | -0.00800784 |
| 481 | 31.8473 | 0.0772484 | 0.0293649 |
| 1489 | 31.9106 | 0.295003 | 0.039419 |
| 6 | 31.9181 | 1.20913 | -0.0204267 |
| 1048 | 31.9968 | 5.06968 | 0.0682652 |
| 602 | 32.0267 | 0.079112245 | 0.00719495 |
| 1124 | 32.0291 | 2.59443 | 0.0139462 |
| 1157 | 32.0491 | 0.83078 | -0.0872251 |
| 9 | 32.0729 | 0.956442 | -0.0627224 |
| 10 | 32.167 | 0.108429 | 0.0623863 |
| 1311 | 32.2314 | 1.01911 | -0.00848792 |
| 996 | 32.2647 | 1.27237 | -0.00250281 |
| 623 | 32.2751 | 0.878281 | 0.0215091 |
| 1020 | 32.3051 | 0.0166803 | 0.0734801 |
| 878 | 32.3914 | 0.0709801 | 0.0130513 |
| 1176 | 32.4672 | 4.53938 | 0.0143067 |
| 403 | 32.4825 | 0.830669 | 0.00273502 |
| 585 | 32.5721 | 3.73573 | -0.029071 |
| 598 | 32.5878 | 0.509108 | -0.0473465 |
| 197 | 32.5985 | 0.0430406 | -0.0849389 |
| 21 | 32.6544 | 0.00245146 | 0.0287373 |
| 256 | 32.655 | 0.205801 | -0.00917728 |
| 540 | 32.714 | 2.60542 | 0.0306973 |
| 861 | 32.775 | 2.42819 | -0.0319636 |
| 122 | 32.8604 | 3.14589 | 0.026936 |
| 90 | 32.8978 | 0.540278 | 0.00509936 |
| 539 | 32.9048 | 0.12395 | -0.0475372 |
| 106 | 32.9359 | 0.758391 | -0.00426101 |
| 632 | 32.9479 | 1.49049 | 0.066547 |
| 738 | 32.9749 | 1.83776 | 0.0534686 |
| 325 | 32.982 | 0.0136822 | 0.0326083 |
| 50 | 33.0361 | 0.38049 | 0.0643625 |
| 1129 | 33.0556 | 1.99972 | -0.00246406 |
| 930 | 33.0927 | 0.914435 | -0.0217924 |
| 844 | 33.1288 | 2.68361 | 0.0443689 |
| 932 | 33.1586 | 0.179848 | -0.0213756 |
| 482 | 33.1795 | 2.94302 | -0.0520544 |
| 855 | 33.2364 | 1.64741 | 0.0216558 |
| 370 | 33.2789 | 0.281334 | -0.0150418 |
| 831 | 33.3099 | 3.23663 | 0.0489841 |
| 349 | 33.3102 | 1.09408 | 0.0218587 |
| 505 | 33.367 | 0.74015165 | -0.08633665 |
| 125 | 33.3922 | 0.0952819 | 0.0588993 |
| 192 | 33.4076 | 1.06129 | -0.0200802 |
| 1043 | 33.4459 | 0.227586 | 0.0111065 |
| 4 | 33.4537 | 0.369525 | -0.0761173 |
| 618 | 33.5116 | 3.36029 | -0.0415092 |
| 1371 | 33.5501 | 0.407499 | -0.0408388 |
| 473 | 33.6395 | 1.97883 | 0.081322 |
| 412 | 33.6426 | 0.796649 | -0.0420445 |
| 558 | 33.6519 | 0.438223 | 0.0971012 |
| 447 | 33.7787 | 0.42669 | 0.0116203 |
| 104 | 33.8109 | 1.5966 | 0.115439 |
| 698 | 33.8113 | 0.320759 | 0.0817043 |
| 344 | 33.8582 | 0.15942 | -0.0302706 |
| 549 | 33.8631 | 0.252363 | 0.0125685 |
| 120 | 33.9399 | 2.01623 | -0.0341907 |
| 176 | 33.9756 | 0.851203 | -0.00587281 |
| 63 | 33.9889 | 0.493749 | -0.00933712 |
| 428 | 34.0032 | 0.702387 | -0.0384853 |
| 282 | 34.0196 | 2.62214 | -0.019702 |
| 408 | 34.0699 | 0.0482298 | 0.0757719 |
| 641 | 34.0892 | 0.28779 | 0.0113758 |
|  |  |  |  |
| 89 | 34.1156 | 1.62025 | 0.047653 |
| 31 | 34.1494 | 0.599247 | 0.0617013 |
