# Supplementary material for: Genomic hotspots of chromosome rearrangements explain conserved synteny despite high rates of chromosome evolution in a holocentric lineage
Source: Mol Ecol. 2023 Jul 24;33(24):e17086. doi: 10.1111/mec.17086 (PMC11628656; doi:10.1111/mec.17086)
Supplement: Supplementary file 6 — Table S2. [file MEC-33-e17086-s004.docx]

Table S2. AIC and estimated parameter for chromEvol models. All parameter (excluding BaseN which a haploid chromosome number) are in events per million year.

| AIC | ConFus | LinFus | ConFis | LinFis | BaseR | BaseN | Demi | Dup |
| --- | --- | --- | --- | --- | --- | --- | --- | --- |
| 3607.22 | 0.5251 | 0.0514 | 0.6363 | 0.0150 | 0.0005 | 20 | 0.0052 | 0.0007 |
