# Supplementary material for: Genomic hotspots of chromosome rearrangements explain conserved synteny despite high rates of chromosome evolution in a holocentric lineage
Source: Mol Ecol. 2023 Jul 24;33(24):e17086. doi: 10.1111/mec.17086 (PMC11628656; doi:10.1111/mec.17086)
Supplement: Supplementary file 7 — Table S3. [file MEC-33-e17086-s002.docx]

**Table S2**. Results from glm analysis. The estimate of the coefficient, standard error, z value and probability (Pr) are indicated.

| Coefficient | Estimate | Std. Error | z value | Pr |
| --- | --- | --- | --- | --- |
| *C. little-dalei* |  |  |  |  |
| Intercept | -4.25204 | 2.02576 | -2.099 | 0.0358 |
| satDNA | 0.00351 | 0.00664 | 0.529 | 0.5969 |
| *gypsy* | *0.02511* | *0.01495* | *1.680* | *0.0930* |
| copia | -0.00318 | 0.01391 | -0.229 | 0.8191 |
| class II | 0.04435 | 0.04073 | 1.089 | 0.2763 |
| non LTR | 0.05858 | 0.03740 | 1.566 | 0.1173 |
| Genes | 0.02340 | 0.01824 | 1.283 | 0.1996 |
| *C. cristatella* |  |  |  |  |
| Intercept | -2.712036 | 4.328442 | -0.627 | 0.5309 |
| **satDNA** | **0.001905** | **0.000923** | **2.063** | **0.0391** |
| gypsy | 0.023046 | 0.044729 | 0.515 | 0.6064 |
| copia | -0.043106 | 0.027829 | -1.549 | 0.1214 |
| class II | 0.005125 | 0.029794 | 0.172 | 0.8634 |
| non LTR | -0.000565 | 0.010610 | -0.053 | 0.9575 |
| Genes | 0.000392 | 0.001026 | 0.382 | 0.7026 |
| *C. scoparia* |  |  |  |  |
| Intercept | -14.24 | 7.693 | -1.851 | 0.0641 |
| **satDNA** | **0.004399** | **0.001997** | **2.203** | **0.0276** |
| gypsy | -0.01007 | 0.06151 | -0.164 | 0.8699 |
| *copia* | *0.06429* | *0.03475* | *1.850* | *0.0643* |
| class II | 0.0004185 | 0.05746 | 0.007 | 0.9942 |
| non LTR | -0.2624 | 0.1694 | -1.549 | 0.1214 |
| *Genes* | *0.003031* | *0.001775* | *1.707* | *0.0877* |
